# Theoretical Prediction of the Electronic Properties of Bidentate Ligands (HEP2) and Synthesis of Bis(*N*‑heterocyclic carbene) Silver and Palladium Complexes

**DOI:** 10.1021/acs.inorgchem.5c01231

**Published:** 2025-07-16

**Authors:** Carlos J. Carrasco, Francisco Montilla, Eleuterio Álvarez, Agustín Galindo

**Affiliations:** † Departamento de Química Inorgánica, Facultad de Química, 16778 Universidad de Sevilla, 41012 Sevilla, Spain; ‡ Instituto de Investigaciones Químicas, CSIC-Universidad de Sevilla, Avda. Américo Vespucio 49, 41092 Sevilla, Spain

## Abstract

A theoretical approach based on density functional theory (DFT) was developed to determine the electronic Huynh parameter for bidentate ligands (HEP2). The ^13^C chemical shift of the carbene carbon atom in the 1,3-diisopropylbenzimidazolin-2-ylidene ligand (^i^Pr_2_-bimy) was calculated within the model complexes [PdBr­(L_2_)­(^i^Pr_2_-bimy)]^+^, where L_2_ represents a bidentate ligand. Strong correlations were observed between the calculated and experimental chemical shifts for both *N*-donor and *C*-donor L_2_ ligands, with *R* ^2^ values of 0.9699 and 0.9926, respectively. This DFT approach demonstrates the potential for estimating the HEP2 parameter of bidentate ligands of these types. This method was applied to estimate the HEP2 values for three bis­(*N*-heterocyclic carbene) ligands containing carboxylate groups, (diNHC^R^)^2–^, which are explored as ligands in this study. These NHC ligands were derived from three precursor compounds: 1-(carboxymethyl)-3-((3-(carboxymethyl)-1*H*-imidazol-3-ium-1-yl)­methyl)-1*H*-imidazol-3-ium bromide (**1a**), 1-(1-carboxyethyl)-3-((3-(1-carboxyethyl)-1*H*-imidazol-3-ium-1-yl) methyl)-1*H*-imidazol-3-ium bromide (**1b**), and 2-(1-(2-(1-(carboxymethyl)-1*H*-imidazol-3-ium-3-yl)­ethyl)-1*H*-imidazol-3-ium-3-yl)­acetate bromide (**1c**). They were synthesized and characterized using IR, NMR, and single-crystal X-ray crystallography (for **1a** and **1c**). The interaction of **1a** and **1c** with Ag_2_O in the presence of aqueous sodium hydroxide produced the complexes [Na_2_(H_2_O)_8_]­[Ag_2_(di_CH2_NHC^H^)_2_] (**2a**) and [Na_2_(H_2_O)_8_]­[Ag_2_(di_C2H4_NHC^H^)_2_] (**2c**), respectively. Complex **2a** was structurally characterized, revealing a binuclear structure in which the bis-carbene ligand adopts a bridging μ-κ^1^(C),κ^1^(C′) coordination. Furthermore, **2a** exhibited reactivity in transmetalation, allowing the synthesis of complex [Na_2_(H_2_O)_10_]­[Pd­(di_CH2_NHC^H^)_2_] (**3a**) through a reaction with palladium acetate. Complex **3a**, which was also structurally characterized, consists of a mononuclear tetracarbene species where each bis-carbene ligand coordinates in a bidentate κ^2^(C,C′) fashion.

## Graphical abstract

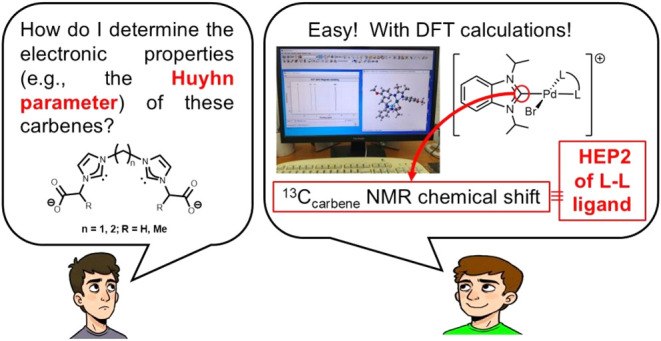

## Introduction

*N*-Heterocyclic carbenes (NHCs) are versatile ligands widely recognized for their stable coordination with metals, leading to a wide range of applications in various fields.^1–5^ The stability of metal-NHC complexes is due to the strong ligand-to-metal donation, which makes the study of their electronic properties critical, particularly in the context of transition metal catalysis. Particularly, polydentate NHCs are designed to enhance the stability of transition metal complexes through the chelating effect. In addition, key factors such as steric hindrance, electronic character, and bite angle can be finely tuned by altering substituents on the nitrogen atom, the backbone, or modifying the linker chain, respectively.^6,7^ These modifications enable precise control of the electronic and steric environment of the metal, with profound implications for catalytic efficiency.^8,9^ Bidentate bis-NHCs typically consist of two imidazole-2-ylidene rings linked by a hydrocarbon chain or heteroatom, (NHC)_2_X.^7^ These ligands generally coordinate in a bidentate κ^2^(C,C′) or a bridging μ-κ^1^(C),κ^1^(C′) fashion. The coordination dichotomy, chelating versus bridging, has been extensively analyzed by Peris and co-workers.^9^

The electronic properties of monodentate ligands are typically evaluated using the Tolman electronic parameter (TEP),^10^ a well-established experimental method. TEP values for monodentate NHC ligands are well-documented;^11,12^ however, there is a notable scarcity of both experimental and theoretical data for bidentate ligands.^13–16^ An alternative experimental method to assess a ligand’s electronic properties, introduced by Huynh, involves the use of ^13^C NMR spectroscopy.^12,17^ Huynh’s electronic parameter (HEP) for a ligand L is defined as the ^13^C_carbene_ NMR chemical shift of the ^i^Pr_2_-bimy ligand in *trans*-[PdBr_2_(L)­(^i^Pr_2_-bimy)] (^i^Pr_2_-bimy = 1,3-diisopropylbenzimidazolin-2-ylidene).^18,19^ Several HEP values for NHC ligands are known, and this parameter has also been extended to bidentate ligands as the HEP2 parameter.^20,21^

While methods for determining TEP^10^ and HEP^12^ parameters in monodentate ligands are well established through experimental and theoretical approaches,^22^ this is not the case for bidentate ligands, where available data are largely limited to HEP2 values.^20,21^ As a result, the development of a theoretical procedure to estimate electronic parameters for bidentate ligands is highly desirable. In this study, we address this gap by developing a density functional theory (DFT) procedure to theoretically determine the HEP2 values. This approach involves calculating the ^13^C chemical shift of C_carbene_ of the ^i^Pr_2_-bimy ligand in models of [PdBr­(L_2_)­(^i^Pr_2_-bimy)]^+^, where L_2_ represents a bidentate ligand. Good correlations have been established between experimental and theoretical HEP2 values for L_2_ ligands with *N*- and *C*-donors, allowing estimation of the electronic parameters for the bis-NHC ligands studied here (Scheme 1). While our research was in progress, a DFT analysis of the NMR characteristics of NHC compounds and the calculation of the HEP parameter was published.^23^ Based on our ongoing research on transition metal complexes containing amino acid-derived imidazolium-carboxylate ligands^24–28^ and related carboxylate-NHC complexes,^29,30^ we expanded our focus to bis­(*N*-heterocyclic carbenes) featuring dicarboxylate moieties, (diNHC^R^)^2–^ (Scheme 1). In this work, we report the synthesis and characterization of the precursor compounds **1a**–**c**, as well as the synthesis and structural characterization of new bis-carbene silver [Na_2_(H_2_O)_8_]­[Ag_2_(di_CH2_NHC^H^)_2_] (**2a**) and [Na_2_(H_2_O)_8_]­[Ag_2_(di_C2H4_NHC^H^)_2_] (**2c**), and palladium [Na_2_(H_2_O)_10_]­[Pd­(di_CH2_NHC^H^)_2_] (**3a**) complexes, which exhibit different coordination modes: μ-κ^1^(C),κ^1^(C′) for **2a** and κ^2^(C,C′) for **3a**.

**Scheme 1:**
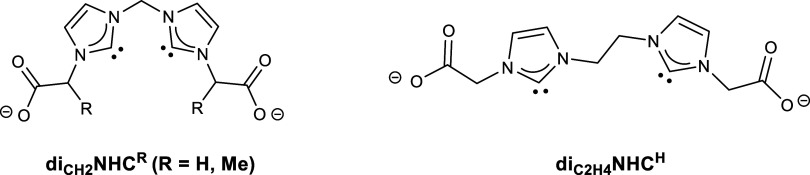
Bis­(*N*-heterocyclic carbene) Ligands Used in this Work

## Results and Discussion

### Synthesis and Characterization of the Precursors of Bis­(carbene) Ligands

For the preparation of ligand precursors **1a** and **1b**, we selected the *N*-alkylation of bis­(imidazole-1-yl)­methane with bromoacetic acid and 2-bromopropionic acid, respectively (Scheme 2). This synthetic approach follows experimental procedures similar to those reported by Santini,^31^ Hemmert,^32^ and de Jesús^33^ for related precursors. We also tried to synthesize the chiral version of **1b** using *S*-alanine and the method developed by Strassner.^34^ However, these attempts were unsuccessful, likely due to the presence of carboxylic groups, which in Strassner compounds were previously esterified.^34^ Compound **1c** was prepared in a similar fashion, using bis­(imidazole-1-yl)­ethane and bromoacetic acid (Scheme 2).

**Scheme 2:**
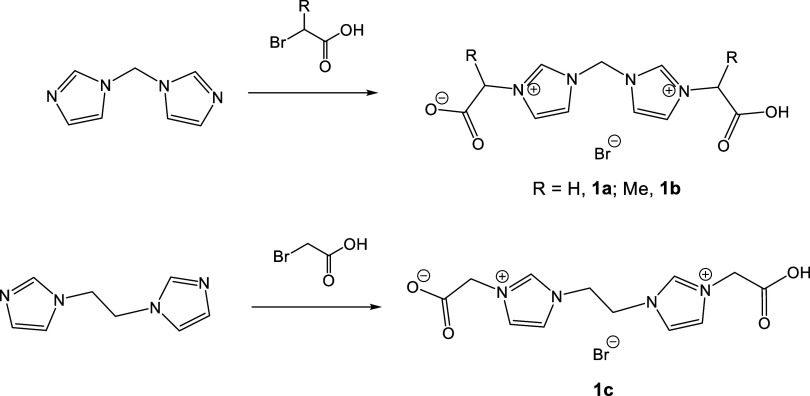
Synthesis of Bis­(carbene) Ligand Precursors

Compounds **1a**–**c** were isolated as hygroscopic white solids, soluble in water and methanol but insoluble in nonpolar solvents. The presence of carboxylate groups in these compounds is confirmed by broad and intense IR bands between 1720 and 1740 cm^–1^, corresponding to antisymmetric ν­(COOH) absorption, and by ^13^C NMR singlets in the range of 170–174 ppm (Figure S1). The methylene bridge in **1a** and **1b** is characterized by NMR signals at approximately 6.7 (^1^H) and 60 (^13^C), while the ethylene bridge in **1c** shows signals at 4.78 (^1^H) and 49.1 ppm (^13^C). The imidazolium rings of **1a**–**c** exhibit the expected NMR pattern, with distinct signals for the nonequivalent C^3^H and C^4^H groups (Figure S1). Crystalline samples of proligands **1a** and **1b** were obtained and structurally characterized by single-crystal X-ray crystallography. A brief description of selected structural parameters (Tables S1 and S2) of these compounds has been included along with the resulting molecular structures (Figures S2–S4).

### Electronic Properties of Bis­(carbene) Ligands

The influence of substituents on the bonding capabilities of carbene-monocarboxylate ligands, [NHC^Ar,R^]^−^, and chiral carbene-dicarboxylate ligands, (*S*,*S*)-NHC^R^ (R = Ala, Val, Leu, Ile), was previously analyzed by us.^29,35^ To further investigate the electronic properties of dianionic bis­(carbene) ligands ((diNHC^R^)^2–^, Scheme 1), we optimized their structures at the mpw1pw91/6-311+G­(d,p) theoretical level (Figure S8 shows the optimized structures). Analysis of the bonding capabilities of the carbenes di_CH2_NHC^H^, di_CH2_NHC^Me^, and di_C2H4_NHC^H^ revealed that the σ orbitals are found in two molecular orbitals (MOs), which are the out-of-phase (HOMO–6) and in-phase (HOMO–7) combinations of σ lone pairs (Figure 1). No significant differences were observed in the MO energies of di_CH2_NHC^R^ ligands with methylene bridges (approximately −1.31 and −1.33 eV for HOMO–6 and HOMO–7, respectively). However, the bis­(NHC) ligand with an ethylene bridge showed a stabilization of approximately 0.11 eV in both MOs, suggesting a weaker σ-donation for this ligand.

**Figure 1:**
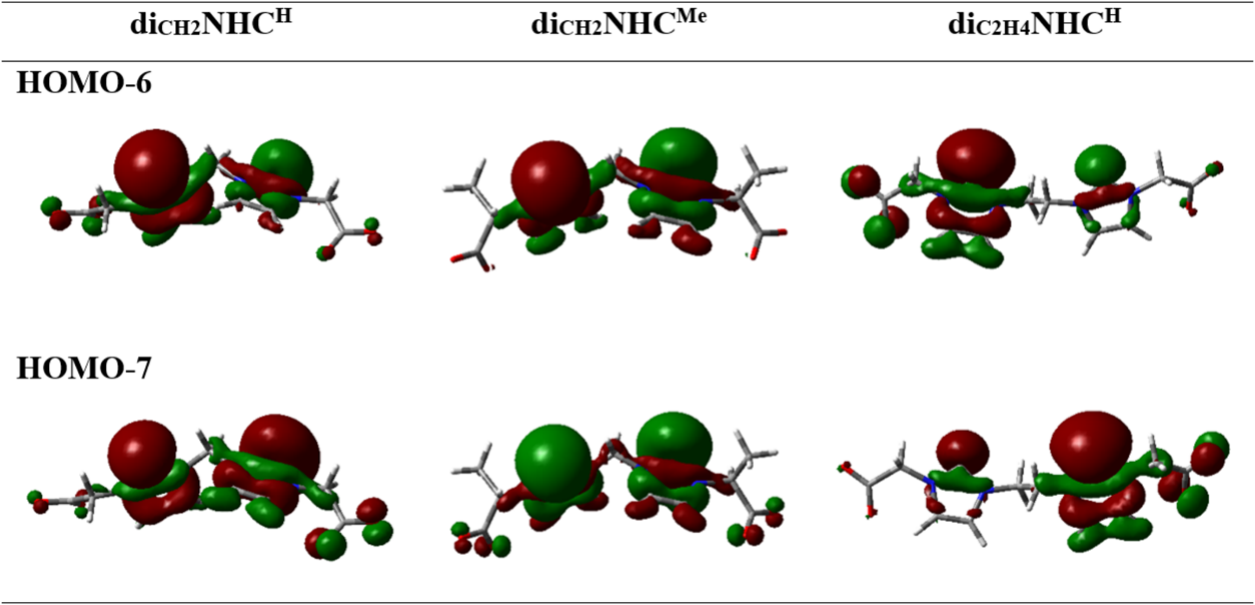
MOs of the σ lone pairs of bis­(carbene) ligands.

To estimate the TEP values for the three bis-carbenes, we used the Gusev approach.^22^ Complexes [Ni­(CO)_3_(κ^1^-diNHC^R^)]^2–^ were optimized with κ^1^-NHC coordination, which is related to the monodentate coordination for each metal observed in the μ-κ^1^(C),κ^1^(C′) bridging mode in silver complex **2a** (see structural characterization below). The calculated ν_CO_ vibration of the A_1_ symmetry was scaled, yielding an estimated TEP value of approximately 2032 cm^–1^ for the three monodentate ligands (diNHC^R^)^2–^ (see Table S7 for additional data). Although a direct comparison is limited due to their potential bidentate nature, this value is similar to those calculated for monodentate NHC ligands with dicarboxylate functionalities (range 2018–2040 cm^–1^),^35^ but lower than the values found for the carbenes commonly encountered IMes and SIMes (2051 and 2052 cm^–1^ in CH_2_Cl_2_, respectively).^11^ We previously attributed this difference to the dianionic nature of these carbenes compared to neutral NHCs.^35^ Additionally, complexes with bidentate NHC coordination of [Ni­(CO)_2_(κ^2^-diNHC^R^)]^2–^ were optimized (Table S8). As expected, the transition from mono- to bidentate coordination in Ni complexes resulted in a decrease in the CO vibrational frequencies of the A_1_ symmetry. The calculated value of around 1964 cm^–1^ for the three bis-NHC ligands is lower than that of several [Ni­(CO)_2_(PR_3_)_2_] complexes (experimental range: 2045–1980 cm^–1^),^36^ indicating that bis-carbenes are stronger electron donors than two phosphane ligands. This value is also lower than those found for [Ni­(CO)_2_(κ^2^-(S)­Poxim)] complexes (range 2042–2047 cm^–1^), where (S)­Poxim is a *N*-phosphine oxide-substituted imidazole-2-ylidene ligand.^37^ To obtain TEP values for the bidentate coordination of the three bis-NHC ligands, we used Crabtree’s correlation, ν_Ni_ = 0.593ν_Mo_ + 871,^14^ by calculating [Mo­(CO)_4_(κ^2^-diNHC^R^)]^2–^ complexes (Table S9). The TEP values, approximately 2062 cm^–1^, for the three bis-NHC ligands compare well with those obtained for ligands such as 1,1′-dimethyl-4,4′-bi-1,2,4-triazol-5,5′-ylidene and 3,3′-dimethyl-bis-imidazole-2,2′-ylidene (2072 and 2067 cm^–1^, respectively) using the same approach.^13^

As an alternative to TEP, the Huynh electronic parameter (HEP) has been extended to a wide range of bidentate ligands (denoted as HEP2).^12,17,19,21,38^ To determine the HEP2 values of our bidentate carbene ligands (diNHC^R^)^2–^ it would be necessary to synthesize the corresponding palladium complexes [PdBr­(κ^2^-diNHC^R^)­(^i^Pr_2_-bimy)]^−^ (^i^Pr_2_-bimy = 1,3-diisopropylbenzimidazolin-2-ylidene) and record their ^13^C NMR spectra. As an alternative, we explored the possibility of predicting the carbene ^13^C chemical shift using DFT methods. We selected 20 bidentate ligands, L_2_, that feature *N*- and *C*-donor atoms (see Scheme 3), for which experimental HEP2 values are available. The corresponding complexes [PdBr­(L_2_)­(^i^Pr_2_-bimy)]^n+^ (L_2_ = bidentate ligand; n = 0,1) were optimized and the ^13^C NMR spectra were calculated (see the Experimental section for details). These theoretical spectra allowed us to compare the calculated ^13^C_carbene_ NMR chemical shift of the ^*i*^Pr_2_-bimy ligand with the corresponding experimental values (Tables 1 and 2). Figures 2 and 3 show good linear correlations between the experimental and calculated HEP2 values for the L_2_ ligands with *N*- and *C*-donors, respectively. For the *N*-donor ligands, the correlation is described by the equation HEP2_calc_ = 0.8391­(HEP2_exp_) + 59.81, with a coefficient of determination *R* ^2^ of 0.9699. The maximum deviation is 1.2 ppm (observed for ligand L_2_4), and the mean absolute deviation is 0.6 ppm. A slightly better agreement was achieved for the *C*-donor ligands, where the regression equation is given by HEP2_calc_ = 1.1982­(HEP2_exp_) – 13.425, with an *R* ^2^ value of 0.9926. In this case, the maximum deviation is 0.4 ppm (for ligand L_2_12) with a mean absolute deviation of 0.2 ppm. For comparison, a previous study reported a mean absolute deviation of 6.4 ppm for theoretical ^13^C NMR chemical shifts across a range of organic compounds using purely computational data at the same theoretical level.^39^ Our lower deviations are attributed to the incorporation of experimental parameters into linear regression models.^39,40^ Using the established relationship for *C*-donor ligands, we estimated the HEP2 values for our dicarbene ligands (di_CH2_NHC^R^)^2–^ to be 180.6 and 180.1 ppm for R = H and Me, respectively. These values are close to that of the ^i^Pr-substituted dicarbene L_2_13 ligand (180.0 ppm). In comparison, the ligand (di_C2H4_NHC^H^)^2–^ exhibits a lower value of 179.7 ppm, which is consistent with the previously discussed stabilization of the MOs associated with σ-donation.

**Figure 2:**
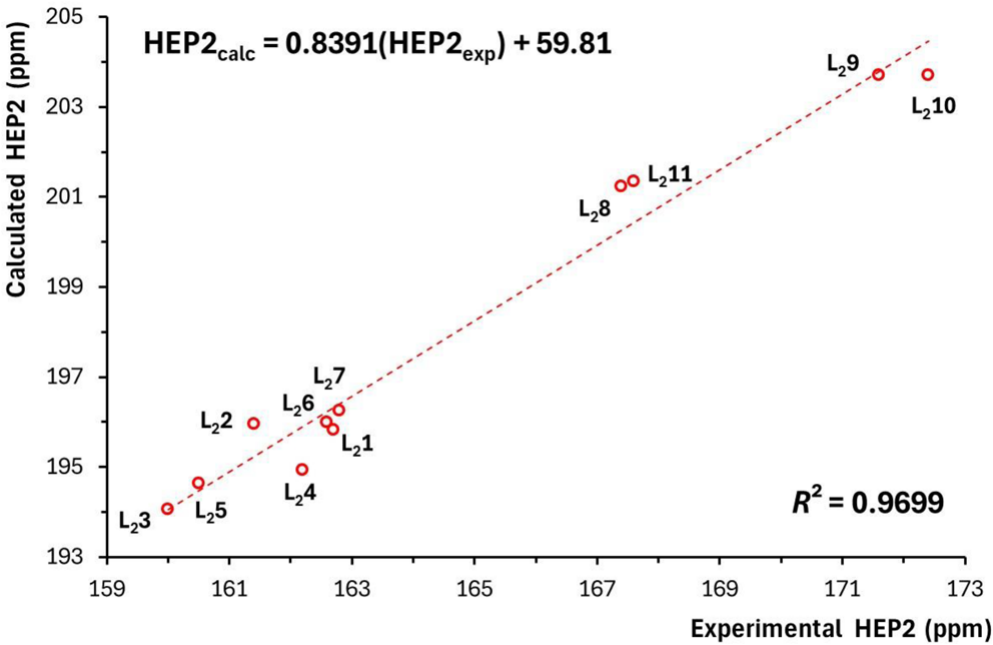
Correlation between the calculated and experimental HEP2 values for selected bidentate *N*-donor ligands.

**Figure 3:**
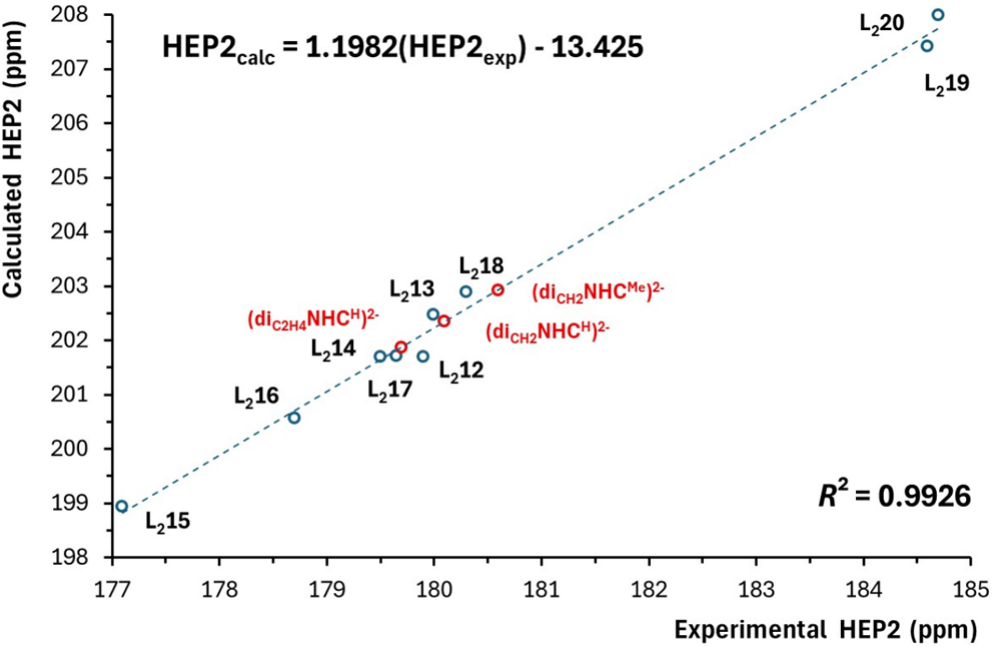
Correlation between the calculated and experimental HEP2 values for the selected bidentate *C*-donor ligands.

**Scheme 3:**
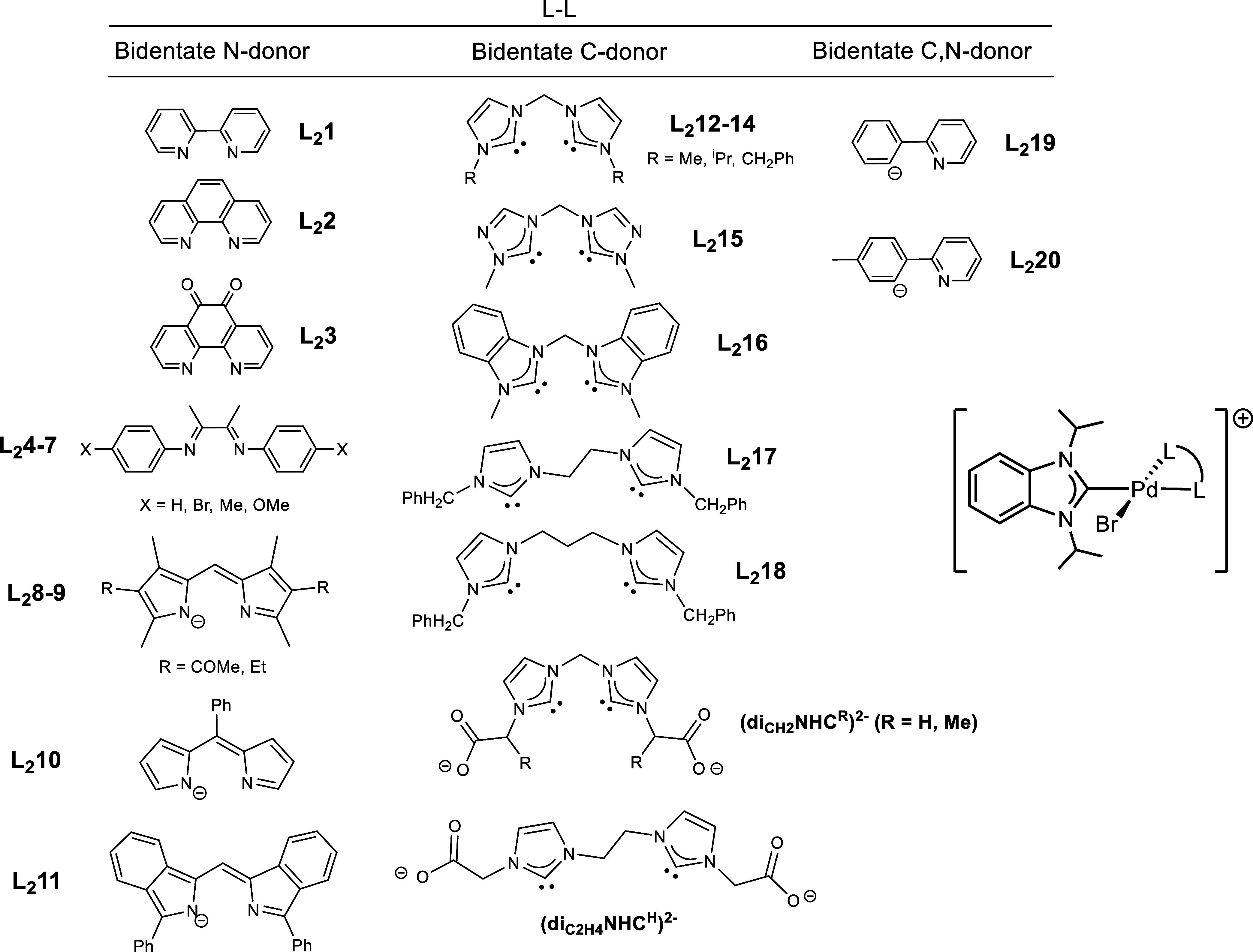
Bidentate L_2_ Ligands for which the HEP2 Values were Theoretically Determined

**Table 1:** Experimental and Calculated HEP2 Values for the Bidentate *N*-Donor Ligands

L_2_ ligand	Experimental HEP2	Calculated HEP2^a^	HEP2 according to correlation^b^	Deviation^c^	References
L_2_1	162.7	195.8	162.1	0.6	^20^
L_2_2	161.4	196.0	162.3	0.9	^20^
L_2_3	160.0	194.1	160.0	0.0	^20^
L_2_4	162.2	194.9	161.0	1.2	^20^
L_2_5	160.5	194.6	160.7	0.2	^20^
L_2_6	162.6	196.0	162.3	0.3	^20^
L_2_7	162.8	196.2	162.6	0.2	^20^
L_2_8	167.4	201.2	168.5	1.1	^41^
L_2_9	171.6	203.7	171.5	0.1	^41^
L_2_10	172.4	203.7	171.5	0.9	^41^
L_2_11	167.6	201.4	168.7	1.1	^41^

a Calculated in this work.

b HEP2_calcd_ = 0.8391­(HEP2_exp_) + 59.81.

c Absolute value.

**Table 2:** Experimental and Calculated HEP2 Values for Bidentate *C*-Donor Ligands

L_2_ ligand	Experimental HEP2	Calculated HEP2^a^	HEP2 according to correlation^b^	Deviation^c^	References
L_2_12	179.9	201.7	179.5	0.4	^20^
L_2_13	180.0	202.5	180.2	0.2	^20^
L_2_14	179.5	201.7	179.5	0.0	^20^
L_2_15	177.1	198.9	177.2	0.1	^20^
L_2_16	178.7	200.6	178.6	0.1	^20^
L_2_17	179.7	201.7	179.6	0.1	^20^
L_2_18	180.3	202.9	180.5	0.2	^20^
L_2_19	184.6	207.4	184.3	0.3	^38^
L_2_20	184.7	208.0	184.8	0.1	^38^
(di_CH2_NHC^H^)^2–^		202.3	180.1		this work
(di_CH2_NHC^Me^)^2–^		202.9	180.6		this work
(di_C2H4_NHC^H^)^2–^		201.9	179.7		this work

a Calculated in this work.

b HEP2_calcd_ = 1.1982­(HEP2_exp_) – 13.425.

c Absolute value.

### Synthesis and Characterization of Silver Complexes **2a** and **2c**

Complexes **2a** and **2c** were synthesized by treating compounds **1a** and **1c**, respectively, with Ag_2_O in the presence of aqueous sodium hydroxide (Scheme 4). They were isolated as colorless crystals or solids in good yields, which were soluble in water and sparingly soluble in methanol but insoluble in other organic solvents. The analogous reaction with **1b** did not afford the corresponding **2b** as a pure complex. Carboxylate groups are characterized by broad intense IR bands at ca. 1600 cm^–1^ (antisymmetric ν­(COO)) and by singlets at 174.6 and 171.1 ppm (for **2a** and **2c**, respectively) in ^13^C NMR spectra (Figure S1). The CH_2_ groups of the bridge resonate in the expected NMR region, for example, at 6.54 and 4.67 ppm (for **2a** and **2c**, respectively) in the ^1^H NMR spectra. The ^13^C NMR signals of the ethylene bridge in **2c** appear at 61.2 ppm (Figure S1), which is shifted with respect to the values found for related benzimidazolium-type bis-NHC silver complexes (44–48 ppm range).^42^ As occurred in related carbene silver complexes, the C_carbene_ signals were not observed in the ^13^C NMR spectra. Although microanalysis suggests a 1:1 ratio for the Ag:carbene ligand, the electrospray ionization mass spectrometry (ESI-MS) spectra indicate a binuclear complex.

**Scheme 4:**
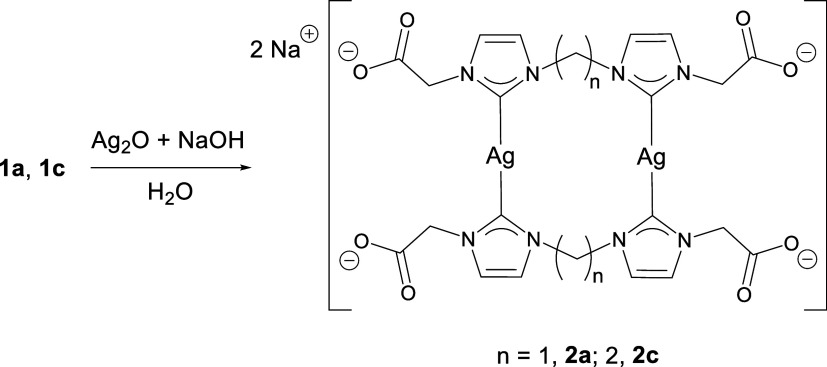
Synthetic Procedure of Complexes **2a** and **2c**

The actual nature of complex **2a** was determined by X-ray crystallography. Single crystals suitable for X-ray diffraction studies of [Na_2_(H_2_O)_8_]­[Ag_2_(di_CH2_NHC^H^)_2_]·4H_2_O, **2a**, were obtained from a concentrated water solution at 0 °C. Figure 4 shows the asymmetric unit of **2a** made up of the anion [Ag_2_(L)_2_]^2–^, the cation [Na_2_(H_2_O)_8_]^2+^, and crystallization water molecules. Selected structural parameters are collected in Table S4. The dimeric anion [Ag_2_(L)_2_]^2–^ displays a “shell-like” structure or “open-book” shape in which each silver ion is coordinated by two carbon atoms of different carbene ligands, μ-κ^1^(C),κ^1^(C′) (Ag–C_carbene_ distances within the range 2.078(8)-2.110(8) Å). The Ag1–Ag2 distance of 3.3794(9) Å is slightly shorter than two times the van der Waals radius of silver (3.44 Å) and suggests a weak intramolecular argentophilic interaction in the solid state. The C_carbene_–Ag–C_carbene_ bond angles deviate from linearity, 166.4(3) and 166.9(3)°, probably due to the latter interaction and the constraint imposed by the methylene group that links the NHC rings. The angle of the “open-book” shape, namely, between the planes defined by the imidazolium rings coordinated to each silver atom, is 74.01° (Figure S5), which is similar, for example, to that reported for [Ag_2_(bisMeOEtIm)_2_]­(NO_3_)_2_ (73.38(7)°).^43^ The N–C_bridge_–N angles of approximately 111° are slightly higher than those observed for the precursor ligand (108.6(7)°) and compare well with other related complexes.^44,45^ The carboxylate groups of ligands show similar C=O bond lengths (range 1.231(11)–1.259(11) Å). They are slightly longer than those observed in the ligand precursors **1a** and **1b**, due to their involvement in the interaction with sodium cations (Na1–O5, 2.343(7) Å; Na1–O1, 2.621(8) Å) and in the formation of hydrogen bonds with molecules of hydration. In fact, the hydrogen bonding network controls the three-dimensional (3D) packing of **2a**, where the two carboxylate groups of each ligand are oriented to the same part, one pair toward the convex part of the “open-book” shape and the other pair toward the concave one (Figure S6).

**Figure 4:**
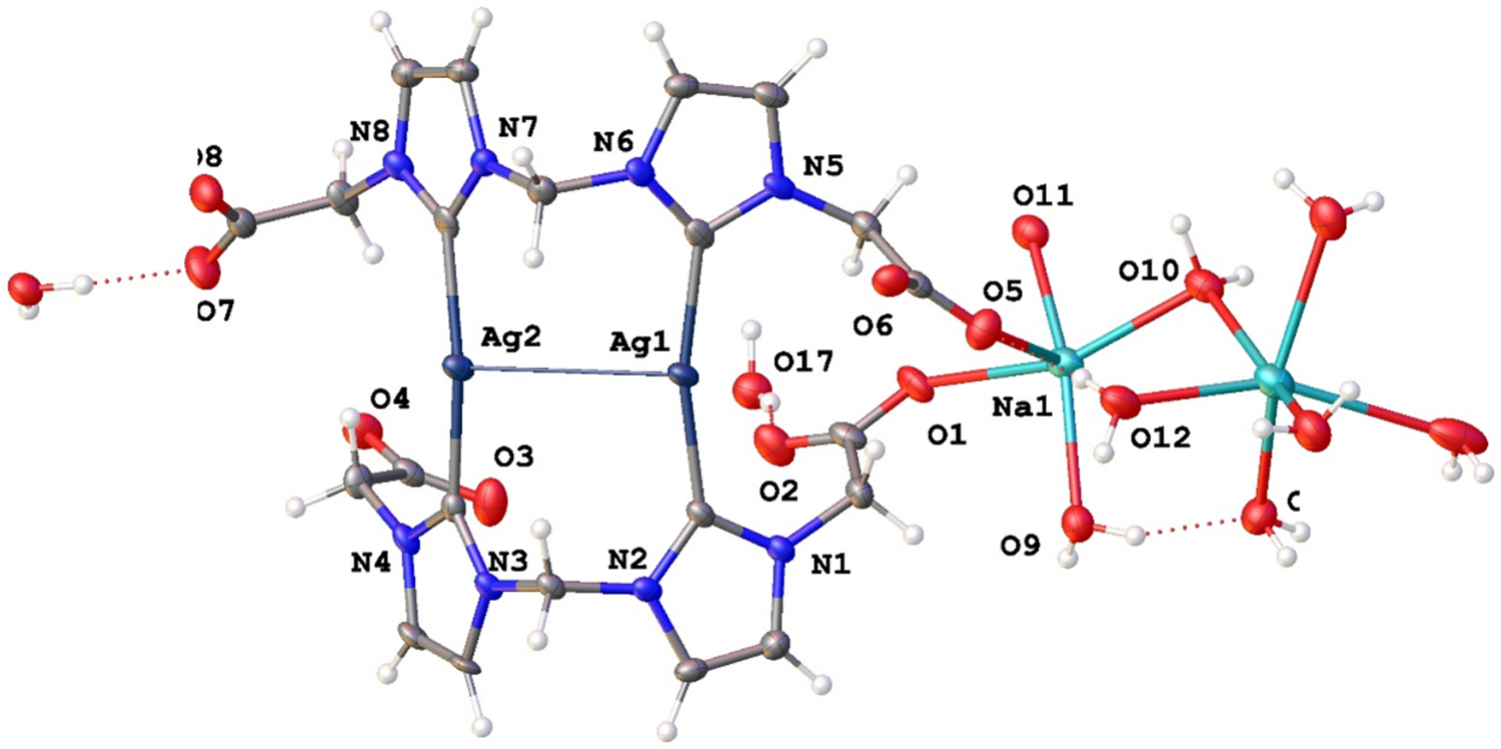
Asymmetric unit of complex **2a**.

### Synthesis and Structure of Palladium Complex **3a**

*In situ* prepared water solution of **2a** was reacted with Pd­(AcO)_2_ to obtain complex **3a** with moderate yields, which were obtained as a colorless solid (Scheme 5). The absorption at 1610 cm^–1^ in the IR spectrum of **3a**, assigned to the antisymmetric mode ν­(COO), is similar to that found for **2a**. However, the binuclear formulation is discarded on the basis of the ESI-MS spectrum of **3a**, which is indicative of a ratio of two carbene ligands for one palladium. A tetracarbene formulation is proposed, similar to those reported by related palladium­(II) complexes.^33,46–49^ The bridging methylene H atoms of **3a** are diastereotopic and appear as an AB system in the typical ^1^H NMR region (6.23 and 6.46 ppm).^33^ A single resonance is found for the carbene carbon atom in the ^13^C NMR spectrum at 171.5 ppm, while the carboxylate carbon atoms appear at 173.6 ppm. These assignments were confirmed by a ^1^H–^13^C­{^1^H} HSQC NMR spectrum (Figure S1).

**Scheme 5:**
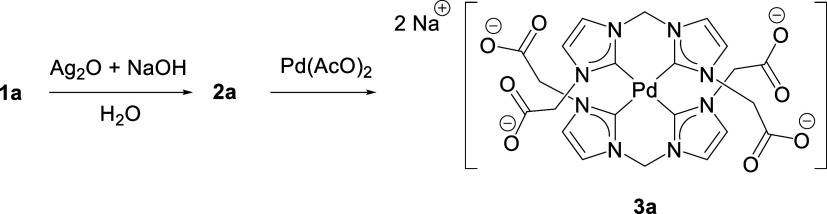
Synthetic Procedure of Complex **3a**

Suitable **3a** crystals were obtained from a concentrated water solution at 0 °C and this complex was identified as [Na_2_(H_2_O)_10_]­[Pd­(di_CH2_NHC^H^)_2_]·3H_2_O. They crystallize in the triclinic system in the centrosymmetric space group *P*1̅. The asymmetric unit of **3a** consists of half of the symmetrically independent dianionic complex [Pd­(di_CH2_NHC^H^)_2_]^2–^ (Pd coincides with a center of symmetry), accompanied by half of the cationic unit [Na_2_(H_2_O)_10_]^2+^ (with a center of symmetry between the two sodium ions) plus three crystallization water molecules. Figure 5 shows the structure of **3a**, while selected bond distances and angles are shown in Table S5. The two bis-carbene ligands are coordinated to the Pd center, which is in a square-planar coordination environment. The Pd–C_carbene_ bond distances (C1–Pd1 = 2.0305(11) and C5–Pd1 = 2.0200(11) Å) match well with related tetracarbene complexes.^33,46–49^ The resulting metallacycle shows a boat conformation in which the angle between the planes defined by the imidazolium rings is 69.54°, while the angle around the methylene bridge is 108.53(10)° (N2–C4_bridge_-N4). Unlike that observed in **2a**, the carboxylate groups do not interact with sodium cations (see Figure 4) but are involved in hydrogen bond interactions with water molecules (hydration molecules of sodium or crystallization, Figure S7).

**Figure 5:**
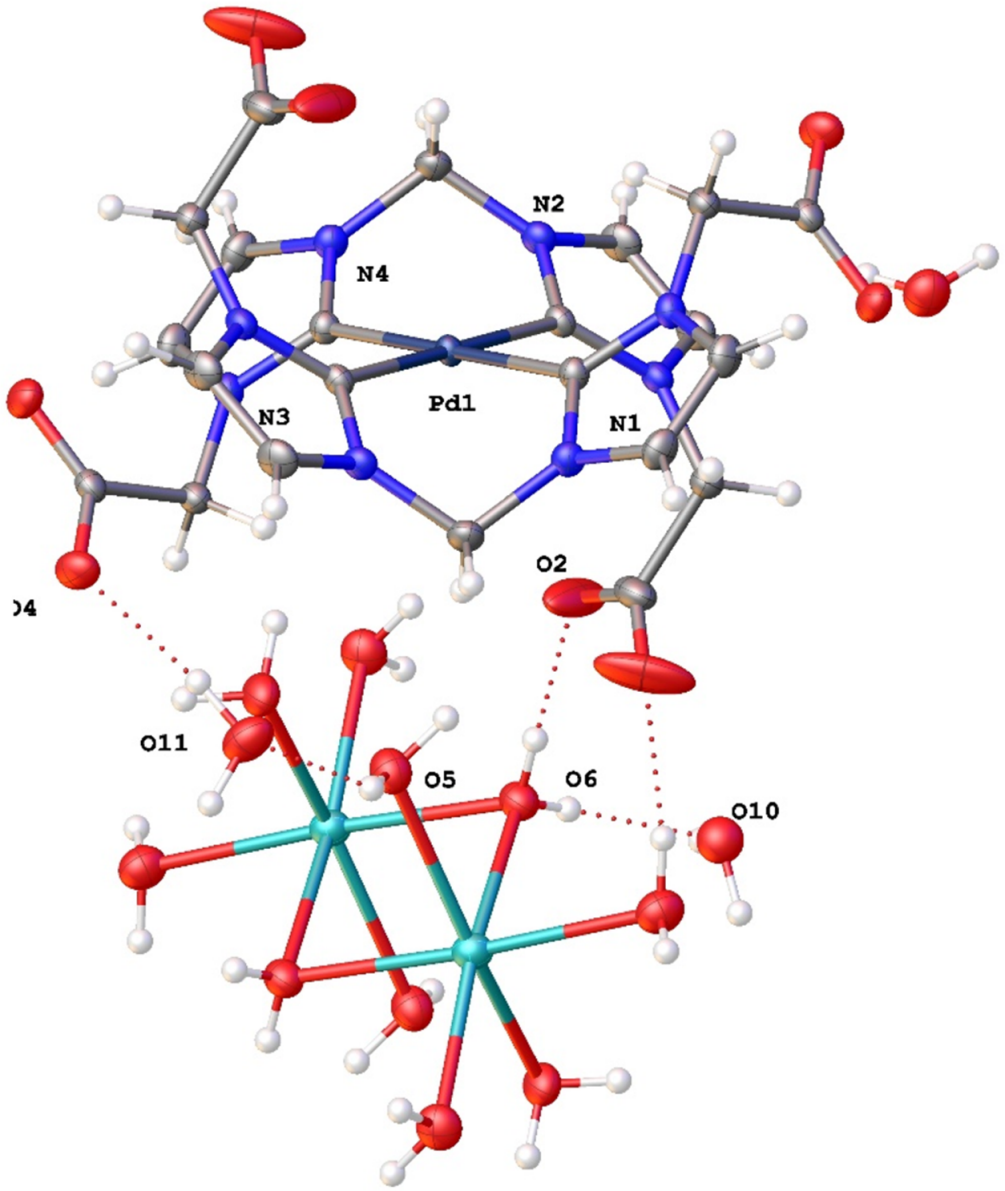
Structure of complex **3a**.

## Conclusions

A theoretical DFT procedure was developed to determine the HEP2 value for bidentate ligands. The calculated ^13^C chemical shift of the carbene carbon atom in the ^i^Pr_2_-bimy ligand within the [PdBr­(L_2_)­(^i^Pr_2_-bimy)]^+^ model complexes (where L_2_ represents a bidentate ligand) shows a good correlation with experimental values for *N*-donor and *C*-donor L_2_ ligands. The *R* ^2^ coefficients of 0.9699 and 0.9926, respectively, were obtained with mean absolute deviations of 0.2 and 0.6 ppm, respectively. Using this methodology, the HEP2 values for the three bis-carbene ligands (diNHC^R^)^2–^ were calculated, falling within the 179–181 ppm range, which represents the upper limit of known experimental HEP2 values of bis-carbene ligands. Experimentally, these bis-carbene ligands are derived from precursors **1a**–**c**, which have been synthesized and characterized. The structures of compounds **1a** and **1c** were determined by X-ray diffraction crystallography. The reaction of **1a** and **1c** with Ag_2_O in the presence of aqueous sodium hydroxide resulted in the formation of binuclear silver complexes **2a** and **2c**, respectively. Complex **2a** was characterized by X-ray crystallography as [Na_2_(H_2_O)_8_]­[Ag_2_(di_CH2_NHC^H^)_2_]·2H_2_O, where the bis-NHC ligand coordinates in a μ-κ1­(C),κ1­(C′) fashion. Complex **3a** was synthesized by the transmetalation reaction of **2a** with palladium acetate and identified as [Na_2_(H_2_O)_10_]­[Pd­(di_CH2_NHC^H^)_2_]·3H_2_O complex, a tetracarbene species in which the bis-NHC ligand coordinates in a bidentate κ^2^(C,C′) fashion.

## Experimental Section

### General

All preparations and other operations were carried out under aerobic (ligand precursors) or anaerobic (complexes) conditions. Solvents were properly purified using standard procedures. The chemicals were obtained from several commercial sources and used as supplied. No uncommon hazards were noted during the synthesis of compounds **1**–**3**. Infrared spectra were recorded using the ATR technique on a PerkinElmer FT-IR Spectrum Two. NMR spectra were recorded at the Centro de Investigaciones, Tecnología e Innovación (CITIUS) of the University of Sevilla using Avance III spectrometers. The ^1^H and ^13^C­{^1^H} NMR shifts were referenced to the residual signals from deuterated solvents. All data are reported in parts per million downfield from Si­(CH_3_)_4_. Elemental analyses (C, H, N) were performed by CITIUS of the University of Sevilla on an Elemental LECO CHNS 93 analyzer. High-resolution mass spectra were obtained on a Q Exactive Hybrid Quadrupole-Orbitrap mass spectrometer from Thermo Scientific (CITIUS of the University of Sevilla).

### Synthesis

#### 1-(Carboxymethyl)-3-((3-(carboxymethyl)-1*H*-imidazol-3-ium-1-yl)­methyl)-1*H*-imidazol-3-ium Bromide (**1a**)

In a round-bottomed flask, bis­(1H-imidazole-1-yl)­methane (750 mg, 5 mmol) and bromoacetic acid (1.528 g, 11 mmol) were added to toluene (40 mL). The mixture was refluxed for 24 h. After this time, it was evaporated to dryness and the resulting solid was washed with 15 mL of MeOH. The remaining solid is taken to dryness to obtain the desired compound. The MeOH extraction was concentrated to half the volume and cooled to −18 °C. An additional crop of 1a was obtained, filtered, and dried. Compound **1a** is obtained as a white powdery solid. Yield: 0.875 g (51%). IR (ATR, cm^–1^): 3066 (w), 1719 (s), 1575 (s), 1542 (s), 1394 (s), 1210 (m), 1166 (s), 856 (w), 761 (m), 650 (m), 616 (m), 417 (w). ^1^H NMR (300 MHz, CD_3_OD): δ 9.31 (s, 2H, N-C*H*_im_-N), 7.82 (s, 2H, C*H*_im_), 7.62 (s, 2H, C*H*_im_), 6.76 (s, 2H, N-C*H*_2_-N), 5.00 (s, 4H, C*H*_2_COO). ^13^C­{^1^H} NMR (75.47 MHz, CD_3_OD): δ 170.6 (*C*OO), 138.2 (N-*C*H_im_-N), 125.0 (*C*H_im_), 121.9 (*C*H_im_), 59.1 (N-*C*H_2_-N), 51.7 (*C*H_2_COO). ESI-MS (negative mode): found *m*/*z* = 263.0782 for **1a**, calculated *m*/*z* = 263.0786 for C_11_H_11_N_4_O_4_. Elemental anal. calcd for C_11_H_15_N_4_O_5_Br (**1a** + H_2_O): C, 36.38; H, 4.16; N, 15.33. Found: C, 35.90; H, 3.85; N, 14.78%.

#### 1-(1-Carboxyethyl)-3-((3-(1-carboxyethyl)-1*H*-imidazol-3-ium-1-yl)­methyl)-1*H*-imidazol-3-ium bromide (**1b**)

Following a similar procedure, **1b** was obtained as a white hygroscopic solid starting from bis­(1H-imidazole-1-yl)­methane and 2-bromopropanoic acid. Yield: 0.750 g (40%). IR (ATR, cm^–1^): 3087 (w), 2972 (w), 1740 (s), 1619 (s), 1161 (s), 1086 (m), 845 (m), 755 (vs), 655 (m), 611 (m). ^1^H NMR (300 MHz, CD_3_OD): δ 9.38 (s, 2H, N-C*H*_im_-N), 7.82 (m, 2H, C*H*_im_), 7.69 (s, H, C*H*_im_), 6.75 (s, 4H, N-C*H*_2_-N), 5.16 (c, 2H, C*H*-CH_3_), 1.79 (s, 4H, C*H*_3_). ^13^C­{^1^H} NMR (75.47 MHz, CD_3_OD): δ 174.1 (*C*OO), 137.1 (N-*C*H_im_-N), 123.7 (*C*H_im_), 121.9 (*C*H_im_), 60.5 (N-*C*H_2_-N), 59.2 (*CH*-CH_3_), 17.5 (*C*H_3_-CH). ESI-MS (negative mode): found *m*/*z* = 291.1095 for **1b**, calculated *m*/*z* = 291.1093 for C_13_H_15_N_4_O_4_. Elemental anal. calcd for C_13_H_23_N_4_O_7_Br (**1b** + 3H_2_O): C, 36.55; H, 5.17; N, 13.11. Found: C, 35.99; H, 4.50; N, 12.41%.

#### 2-(1-(2-(1-(Carboxymethyl)-1*H*-imidazol-3-ium-3-yl)­ethyl)-1*H*-imidazol-3-ium-3-yl)­acetate bromide (**1c**)

Following a similar procedure, **1c** was obtained as a white powdery solid, starting from bis­(1H-imidazole-1-yl)­ethane (162 mg, 1 mmol) and bromoacetic acid (306 mg, 2.2 mmol). Yield: 0.115 g (41%). IR (ATR, cm^–1^): 3134 (w), 3079 (m), 2975 (w), 1726 (s), 1671 (s), 1559 (m), 1477 (w), 1436 (w), 1424 (w), 1392 (w), 1355 (w), 1287 (m), 1159 (s), 1121 (w), 1085 (w), 1043 (w), 927 (w), 866 (m), 797 (s), 767 (s), 681 (m), 655 (m), 638 (s), 518 (w), 497 (w). ^1^H NMR (300 MHz, D_2_O): δ 8.73 (s, 2H, N-C*H*_im_-N), 7.53 (m, 2H, C*H*_im_), 7.52 (s, H, C*H*_im_), 4.92 (s, 4H, C*H*_2_COO), 4.78 (s, 4H, N-C*H*_2_C*H*_2_-N). ^13^C­{^1^H} NMR (75.47 MHz, D_2_O): δ 170.6 (*C*OO), 137.2 (N-*C*H_im_-N), 124.8 (*C*H_im_), 122.1 (*C*H_im_), 51.1 (*C*H_2_COO), 49.1 (N-*C*H_2_ *C*H_2_-N). ESI-MS (negative mode): found *m*/*z* = 277.0938 for **1c**, calculated *m*/*z* = 277.0942 for C_12_H_13_N_4_O_4_. Elemental anal. calcd for C_12_H_17_N_4_O_5_Br (**1c** + H_2_O): C, 38.21; H, 4.54; N, 14.85. Found: C, 37.28; H, 4.51; N, 15.03%.

#### [Na_2_(H_2_O)_8_]­[Ag_2_(di_CH2_NHC^H^)_2_], **2a**

Compounds **1a** (266.66 mg, 1 mmol) and Ag_2_O (116.0 mg, 0.5 mmol) are suspended in deoxygenated H_2_O (5 mL) under a nitrogen atmosphere, and NaOH (80 mg) is added. It is left to react under stirring for 24 h. The resulting reaction mixture is centrifuged. The solution was concentrated under reduced pressure until some precipitation was formed. Then, it was cooled at 0 °C overnight. Complex **2a** is obtained as colorless crystal plates. Yield: 0.280 g (71%). IR (ATR, cm^–1^): 3400 (br), 3157 (m), 3094 (m), 2995 (w), 2905 (w), 1596 (vs), 1517 (w), 1455 (w), 1438 (w), 1420 (w), 1390 (s), 1383 (s), 1302 (s), 1233 (s), 1188 (w), 1168 (w), 1079 (w), 916 (w), 801 (w), 762 (w), 728 (m), 696 (m), 682 (m), 580 (w). ^1^H NMR (300 MHz, CD_3_OD): δ 7.56 (m, 2H, C*H*_im_), 7.30 (s, 2H, C*H*_im_), 6.54 (s, 4H, N-C*H*_2_-N), 4.74 (m, 2H, C*H*_2_COO). ^13^C­{^1^H}-NMR (75.47 MHz, CD_3_OD): δ 174.6 (*C*OO), 123.8 (*C*H_im_), 121.3 (*C*H_im_), 64.4 (N-*C*H_2_-N), 55.0 (*C*H_2_COO). ESI-MS (negative mode): found *m*/*z* = 738.9565 for **2a** + H^+^, calculated *m*/*z* = 738.9517 for C_22_H_21_N_8_O_8_Ag_2_. Elemental anal. calcd for C_22_H_32_N_8_O_14_Na_3_Ag_2_ (2a): C, 27.40; H, 3.97; N, 11.62. Found: C, 28.66; H, 3.16; N, 11.72%.

#### [Na_2_(H_2_O)_8_]­[Ag_2_(di_C2H4_NHC^H^)_2_], **2c**

Following the same method as that in **2a**, using **1c** (92 mg, 0.33 mmol) and Ag_2_O (39 mg, 0.165 mmol) with deoxygenated H_2_O (3 mL) and NaOH (26 mg, 0,66 mmol), **2c** was obtained as a light brown solid. Yield: 0.080 g (60%). IR (ATR, cm^–1^): 3094 (w), 2933 (w), 1600 (vs), 1434 (m), 1385 (s), 1307 (s), 1232 (m), 1186 (w), 1079 (w), 969 (w), 917 (w), 799 (w), 711 (m), 665 (w), 627 (w), 581 (w), 407 (w). ^1^H NMR (500 MHz, D_2_O): δ 7.15 (s, 8H, C*H*_im_), 4.67 (s, 8H, N-CH_2_CH_2_-N), 4.73 (m, 4H, C*H*_2_COO). ^13^C­{^1^H} NMR (125 MHz, D_2_O): δ 171.1 (*C*OO), 123.1 (*C*H_im_), 121.8 (*C*H_im_), 61.2 (N-CH_2_CH_2_-N), 52.2 and 44.7 (*C*H_2_COO).

#### [Na_2_(H_2_O)_10_]­[Pd­(di_CH2_NHC^H^)_2_], **3a**

An aqueous solution of **2a** was prepared as previously stated (using 0.39 mmol of 1a). Over this solution, Pd­(AcO)_2_ (44 mg, 0.19 mmol) was added, and the mixture was left stirring overnight. The mixture was then centrifuged and filtered. Under reduced pressure, the volume was reduced until some precipitation was observed. The yellowish solution was cooled at 0 °C (for 2 weeks). Complex **3a** was obtained as colorless crystal plates. Yield: 0.078 g (61%). IR (ATR, cm^–1^): 3161­(w), 3133­(w), 3021 (w), 1610 (vs), 1561 (s), 1476 (w), 1427 (m), 1398 (s), 1383 (vs), 1319 (m), 1307 (m), 1254 (m), 1233 (m), 1217 (m), 1191 (m), 1171 (m), 1096 (w), 1068 (w), 1040 (w), 998 (w), 969 (w), 920 (w), 812 (m), 795 (w), 762 (m), 729 (s), 700 (m), 676 (s), 582 (w), 579 (m), 532 (w), 472 (w), 430 (m). ^1^H NMR (500 MHz, D_2_O): δ 7.47 (s, 4H, C*H*_im_), 7.10 (s, 4H, C*H*_im_), 6.46 (d, 2H, ^3^ *J*_HH_ = 13.5 Hz, N-C*H*_2_-N), 6.24 (d, 2H, ^3^J_HH_ = 13.5 Hz, N-C*H*_2_-N), 4.33 (d, 4H, ^3^ *J*_HH_ = 17 Hz, C*H*_2_COO), 4.07 (d, 4H, ^3^J_HH_ = 17 Hz, C*H*_2_COO). ^13^C­{^1^H} NMR (CD_3_OD, 75.47 MHz): δ 173.6 (*C*OO), 171.5 (*C*_carbene_), 123.0 (*C*H_im_), 122.2 (*C*H_im_), 63.6 (N-*C*H_2_-N), 53.6 (*C*H_2_COO). ESI-MS (negative mode): found *m*/*z* = 631,0504 for **3a** + H^+^, calculated *m*/*z* = 631.0523 for C_22_H_21_N_8_O_8_Pd.

### Computational Details

The electronic structure and geometries of the di_CH2_NHC^H^, di_CH2_NHC^Me^, and di_C2H4_NHC^H^ carbene ligands were investigated using density functional theory at the mpw1pw91 level^50^ with the 6-311+G­(d,p) basis set. For the calculation of the TEP parameter of carbene ligands, the approach used by Gusev^22^ or Crabtree^14^ was adopted, with the description of the Ni atom using the 6-311+G­(2d) basis set and of the Mo atom using the LANL2DZ basis set.^51^ Molecular geometries were optimized without symmetry restrictions. Frequency calculations were carried out at the same level of theory to identify the stationary points as minima (zero imaginary frequencies). The GIAO method was used for NMR calculations (^13^C NMR isotropic shielding tensors). The mpw1pw91 functional has been recognized as efficient for ^13^C NMR calculations,^52,53^ and optimized structures with this functional were used as input. However, to use the TMS reference that appears in the Gauss View program^54^ the NMR calculation was performed at the B3LYP level^55,56^ with the LANL2DZ basis set^51^ for palladium and the 6-311+G­(2d,p) basis set for other atoms. All theoretical HEP2 values reported in Tables 1 and 2 were calculated in this study. The protocol used for these calculations is included in the Supporting Information. No significant differences were observed when the mpw1pw91 functional was used in the NMR calculations (Figure S9). The density functional theory (DFT) calculations were executed using the Gaussian 09 program package.^57^ The coordinates of all optimized compounds are collected in a separate associated XYZ file attached to the Supporting Information.

### Single-Crystal X-ray Analysis

A summary of the crystallographic data and the structure refinement results for compounds **1a**, **1b**, **2a**, and **3a** is given in Tables S3 and S6. Crystals of a suitable size for the X-ray diffraction analysis were coated with dry perfluoropolyether, mounted on glass fibers, and fixed in a cold nitrogen stream (*T* = 193 K) to the goniometer head. Data collection was carried out on a Bruker-AXS, D8 QUEST ECO, PHOTON II area detector diffractometer, using monochromatic radiation λ­(Mo Kα) = 0.71073 Å, by means of ω and φ scans with a width of 0.50 degrees. Data were reduced (SAINT^58^) and corrected for absorption effects by the multiscan method (SADABS).^59^ Structures were solved by intrinsic phasing modification of direct methods (SHELXT^60^) and refined against all *F* ^2^ data using full-matrix least-squares techniques (SHELXL-2018/3 ^61^), minimizing *w*[*F*_0_^2^–*F*_c_^2^]^2^. All non-hydrogen atoms were refined anisotropically. Hydrogen atoms were included from calculated positions and refined riding on their respective carbon atoms with isotropic displacement parameters. The corresponding crystallographic data were deposited with the Cambridge Crystallographic Data Center as supplementary publications. CCDC 2430657 (**1a**), CCDC 2430658 (**1b**), CCDC 2430659 (**2a**), and CCDC 2430660 (**3a**) contain the supplementary crystallographic data for this paper. The data can be obtained free of charge via: https://www.ccdc.cam.ac.uk/structures/.

## Supplementary Material





## Notes

IR, ^1^H, and ^13^C­{^1^H} NMR spectra of compounds, crystal data and structure refinement crystallographic details, selected structural parameters, structures of **1a** and **1b**, and selected optimized structures from DFT calculations and selected calculated properties (PDF)
Coordinates of optimized structures (XYZ)
