# Supplementary material for: Theoretical Prediction of the Electronic Properties of Bidentate Ligands (HEP2) and Synthesis of Bis(N‑heterocyclic carbene) Silver and Palladium Complexes
Source: Inorg Chem. 2025 Jul 16;64(29):14871–81. doi: 10.1021/acs.inorgchem.5c01231 (PMC12308816; doi:10.1021/acs.inorgchem.5c01231)

Supporting Information

Theoretical prediction of electronic properties of bidentate ligands (HEP2) and synthesis of bis(*N*-heterocyclic carbene) silver and palladium complexes

*Carlos J. Carrasco,[a] Francisco Montilla,[a] Eleuterio Álvarez,[b] and Agustín Galindo[a]**

[a] Departamento de Química Inorgánica, Facultad de Química, Universidad de Sevilla, 41012 Sevilla, Spain.

[b] Instituto de Investigaciones Químicas, CSIC-Universidad de Sevilla, Avda. Américo Vespucio 49, 41092 Sevilla, Spain.

Email address: galindo@us.es (A.G.)

Spectroscopical characterization S3

Figure S1. IR and NMR (^{1}H and ^{13}C{^{1}H}) spectra of compounds **1-3**.

X-ray Crystallography S13

Table S1. Bond lengths and angles for **1a**.

Table S2. Bond lengths and angles for **1b**.

Table S3. Crystal data and structure refinement for **1a** and **1b**.

Figure S2. Structures of compounds **1a** (top) and **1b** (bottom) and their structural description.

Figure S3. Compound **1a**: (up) polymeric chain viewed along *b* axis made up by hydrogen bonds; (b) crystal packing viewed along *b* axis. Color codes: C, grey; H, white; N, blue; O, red; Br, dark red.

Figure S4. Compound **1b**: (up) polymeric chain viewed along *a* axis made up by hydrogen bonds; (b) crystal packing viewed along *c* axis. Color codes: C, grey; H, white; N, blue; O, red; Br, dark red.

Table S4. Selected bond lengths and angles for **2a**.

Table S5. Selected bond lengths and angles for **3a**.

Table S6. Crystal data and structure refinement for **2a** and **3a**.

Figure S5. Front and side views of the anion of **2a**. Color codes: C, grey; H, white; Ag, light grey; N, blue; O, red.

Figure S6. Crystal packing of **2a** along *a*, *b* and *c* axis. Color codes: C, grey; H, white; Ag, cyan; N, blue; Na, light blue; O, red.

Figure S7. Crystal packing of **3a** along *a* axis. Color codes: C, grey; H, white; Pd, blue-gray; N, blue; Na, light blue; O, red.

Theoretical studies (DFT) S26

DFT protocol for estimating HEP2 values.

Figure S8. Optimized structures of bis(carbene) ligands.

Table S7. Optimized structures of $[Ni(CO)_3(diNHC^R)]^{2-}$ complexes and selected calculated properties.

Table S8. Optimized structures of $[Ni(CO)_2(diNHC^R)]^{2-}$ complexes and selected calculated properties.

Table S9. Optimized structures of $[Mo(CO)_4(diNHC^R)]^{2-}$ complexes and selected calculated properties.

Figure S9. Comparison of the calculated ^{13}C NMR chemical shifts using mpw1pw91 and B3LYP functionals.

Figure S1. IR and NMR (^{1}H and ^{13}C{^{1}H}) spectra of compounds **1-3**.

IR spectra

1a

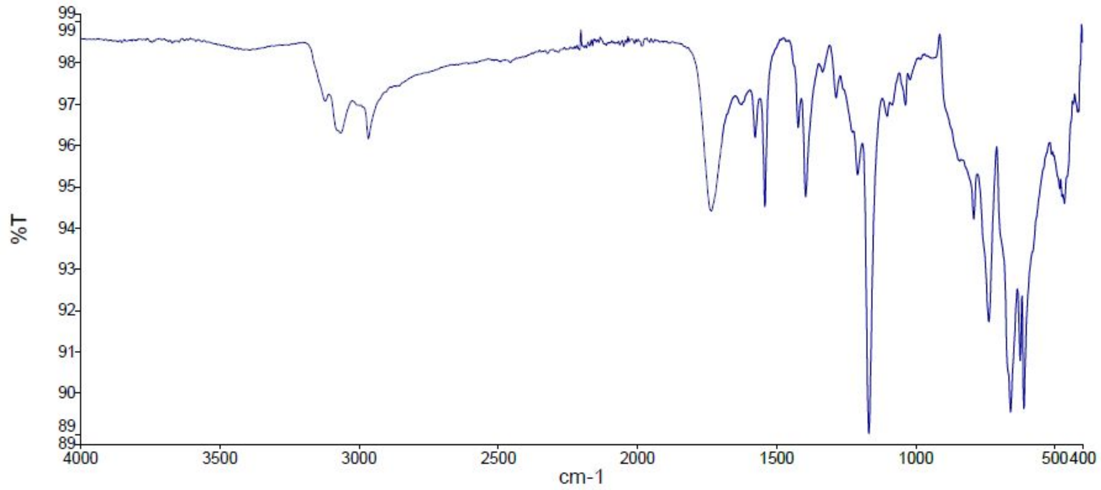

1b

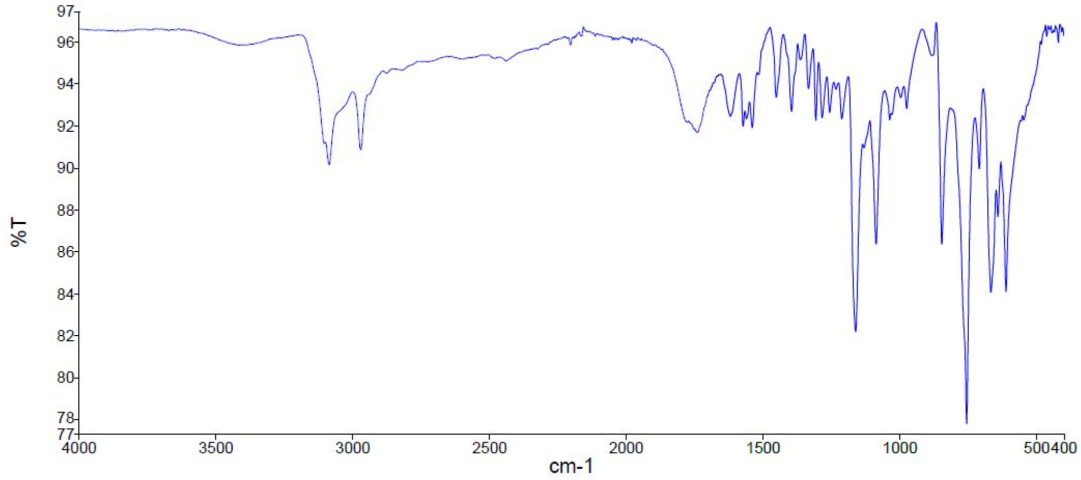

1c

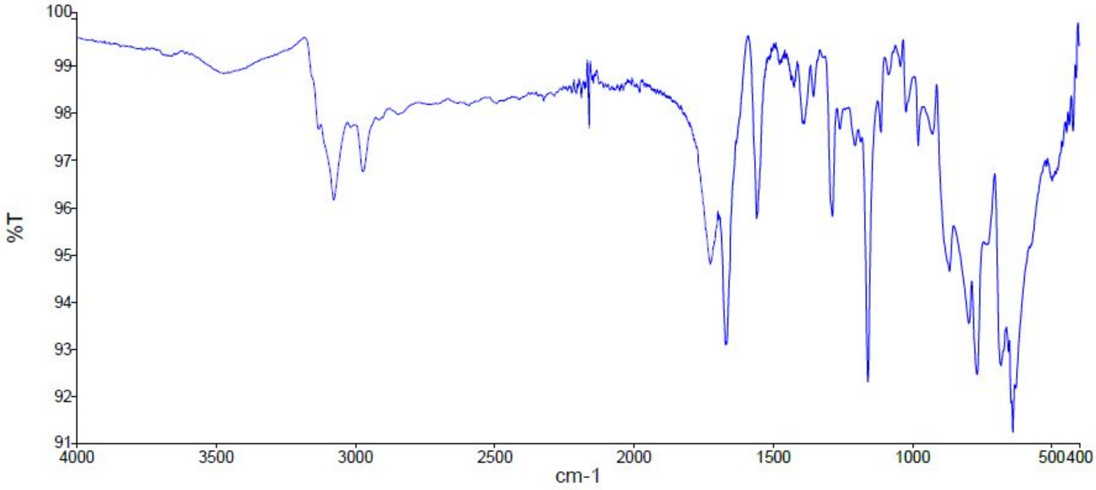
100
99
98
97
96
95
94
93
92
91
%T
4000
3500
3000
2500
2000
1500
1000
500400
cm-1

2a

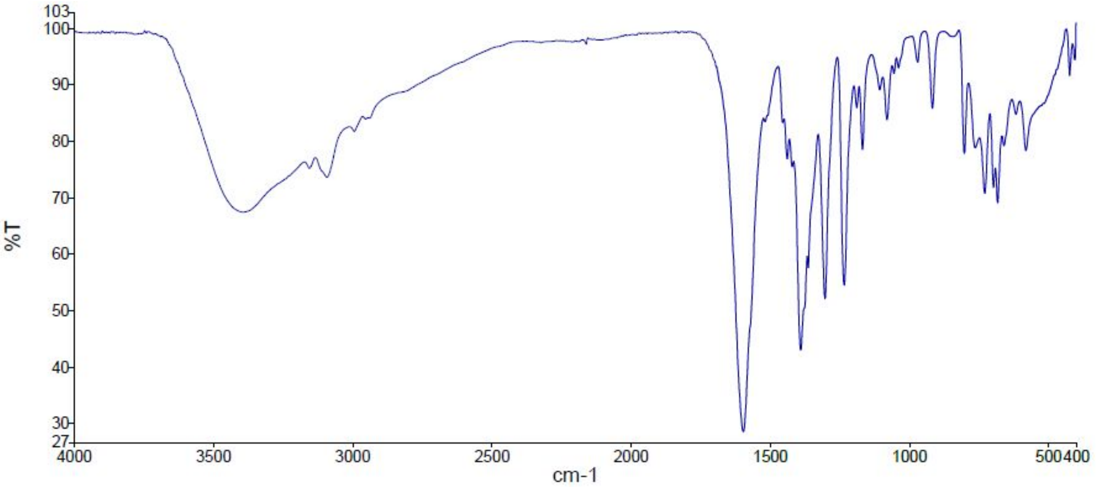
103
100
90
80
70
60
50
40
30
27
%T
4000
3500
3000
2500
2000
1500
1000
500400
cm-1

2c

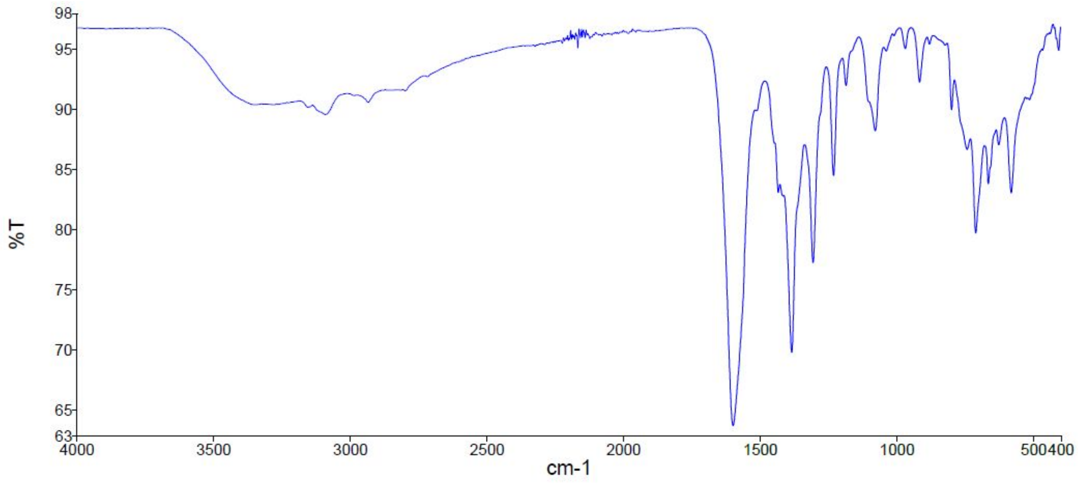

98
95
90
85
80
75
70
65
63
%T
4000
3500
3000
2500
2000
1500
1000
500400
cm-1

3a

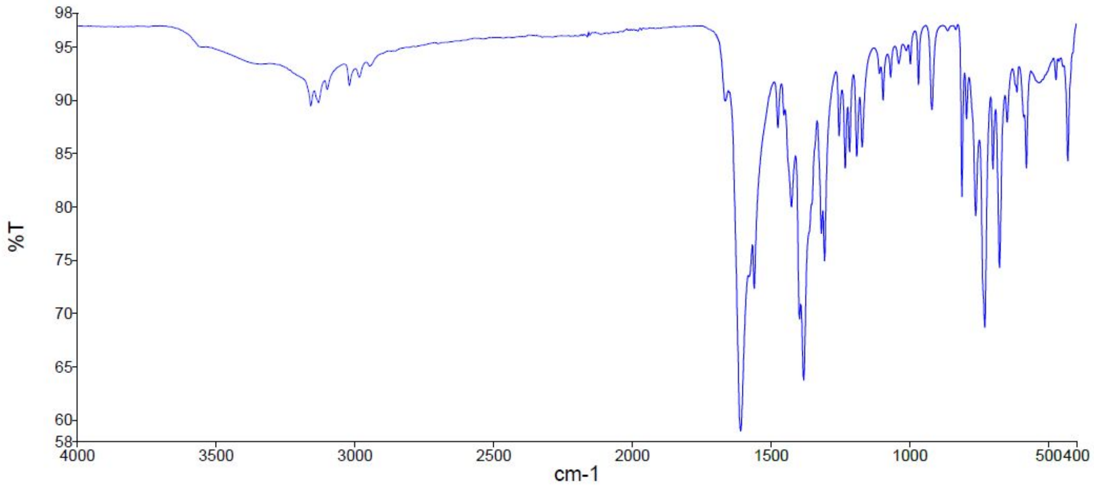

98
95
90
85
80
75
70
65
60
58
%T
4000
3500
3000
2500
2000
1500
1000
500400
cm-1

^{1}H NMR spectra

1a

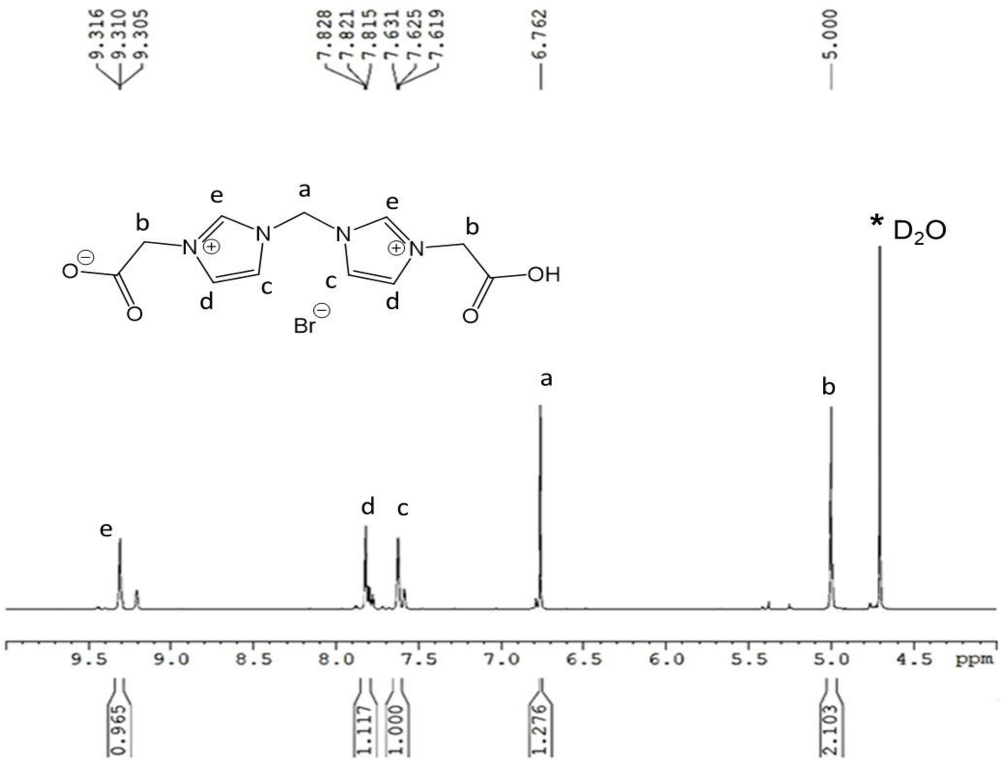

1b

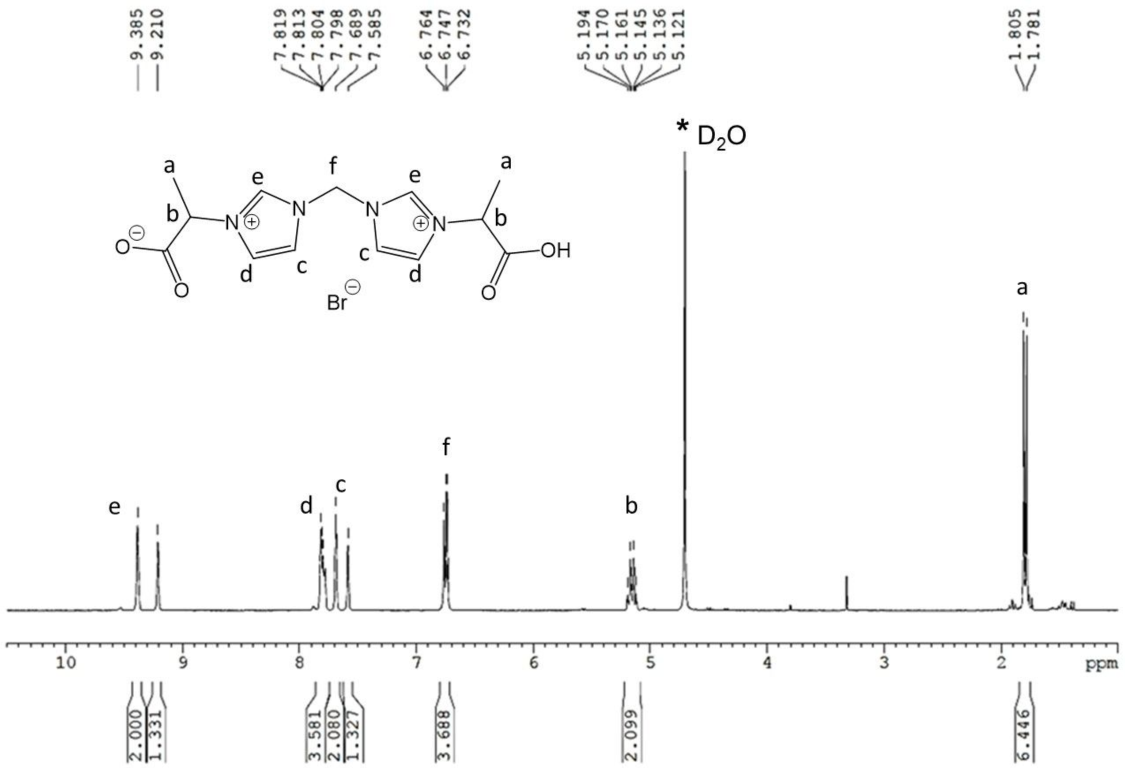

1c

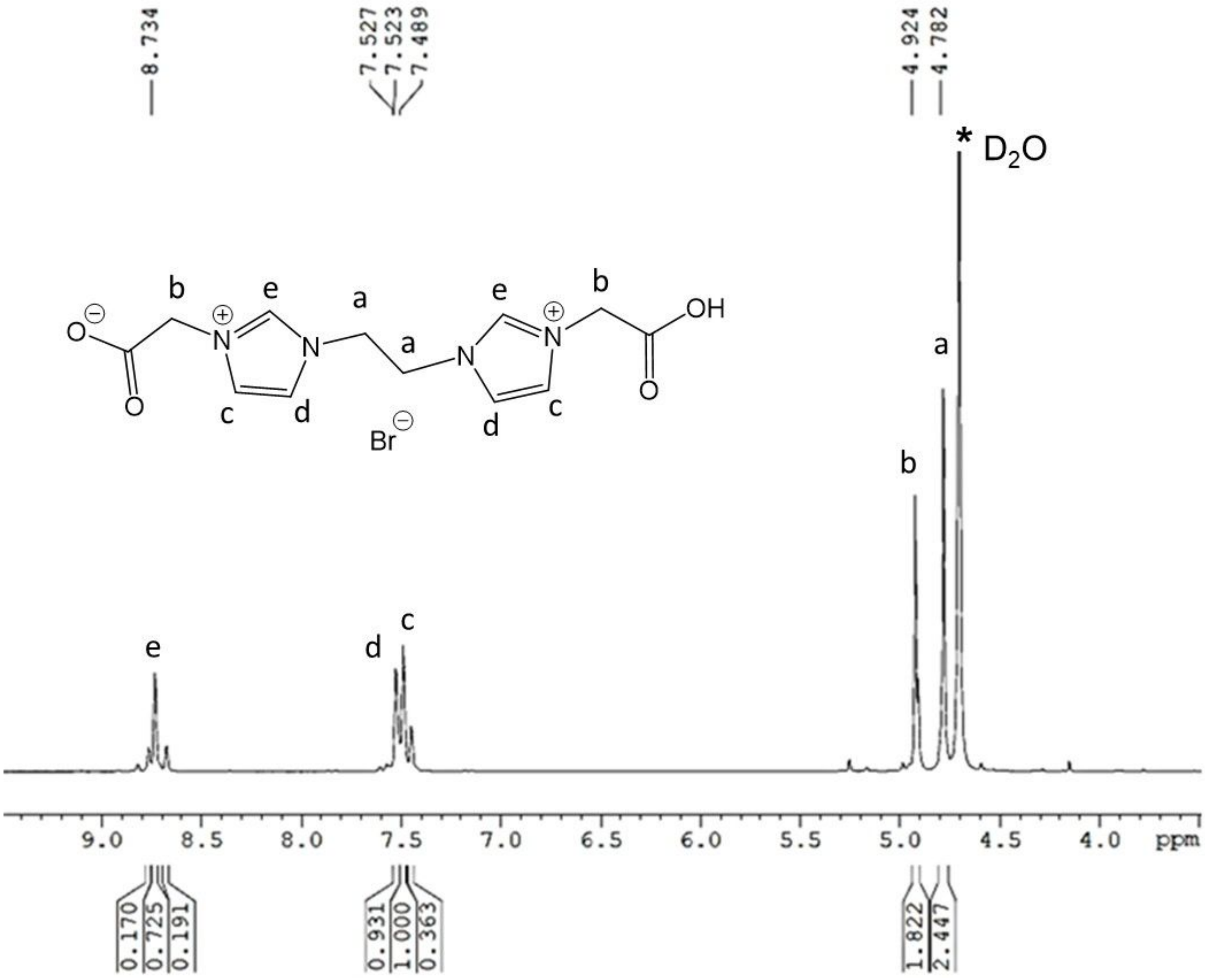

8.734
7.527
7.523
7.489
4.924
4.782
* D2O
b
e
a
a
e
b
OH
O
O
c
d
Br
d
c
a
b
e
d
c
9.0
8.5
8.0
7.5
7.0
6.5
6.0
5.5
5.0
4.5
4.0
ppm
0.170
0.725
0.191
0.931
1.000
0.363
1.822
2.447

2a

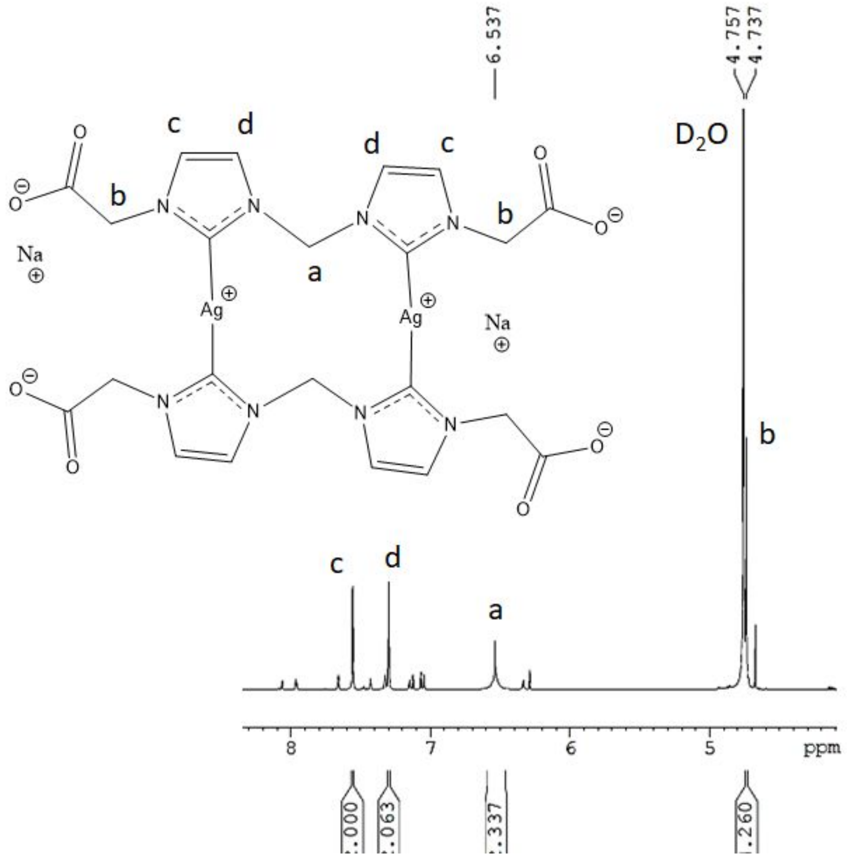

6.537
4.757
4.737
D$_2$O
c
d
a
b
Na
Ag
8
7
6
5
ppm

2c

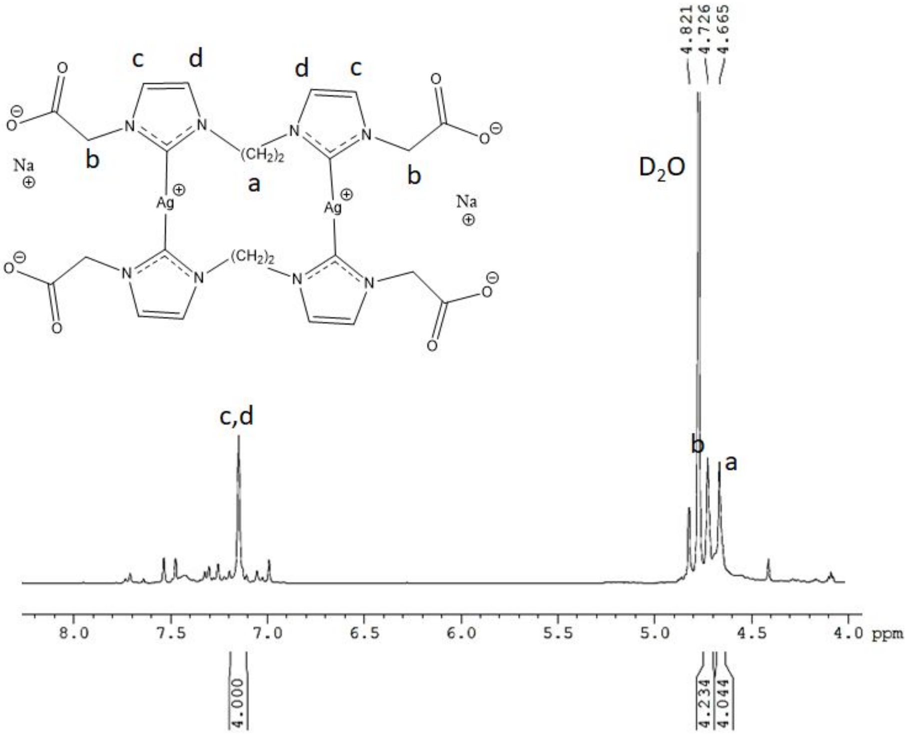

4.821
4.726
4.665
D$_2$O
c,d
b
a
(CH$_2$)$_2$
Na
Ag
8.0
7.5
7.0
6.5
6.0
5.5
5.0
4.5
4.0 ppm
4.000
4.234
4.044

3a

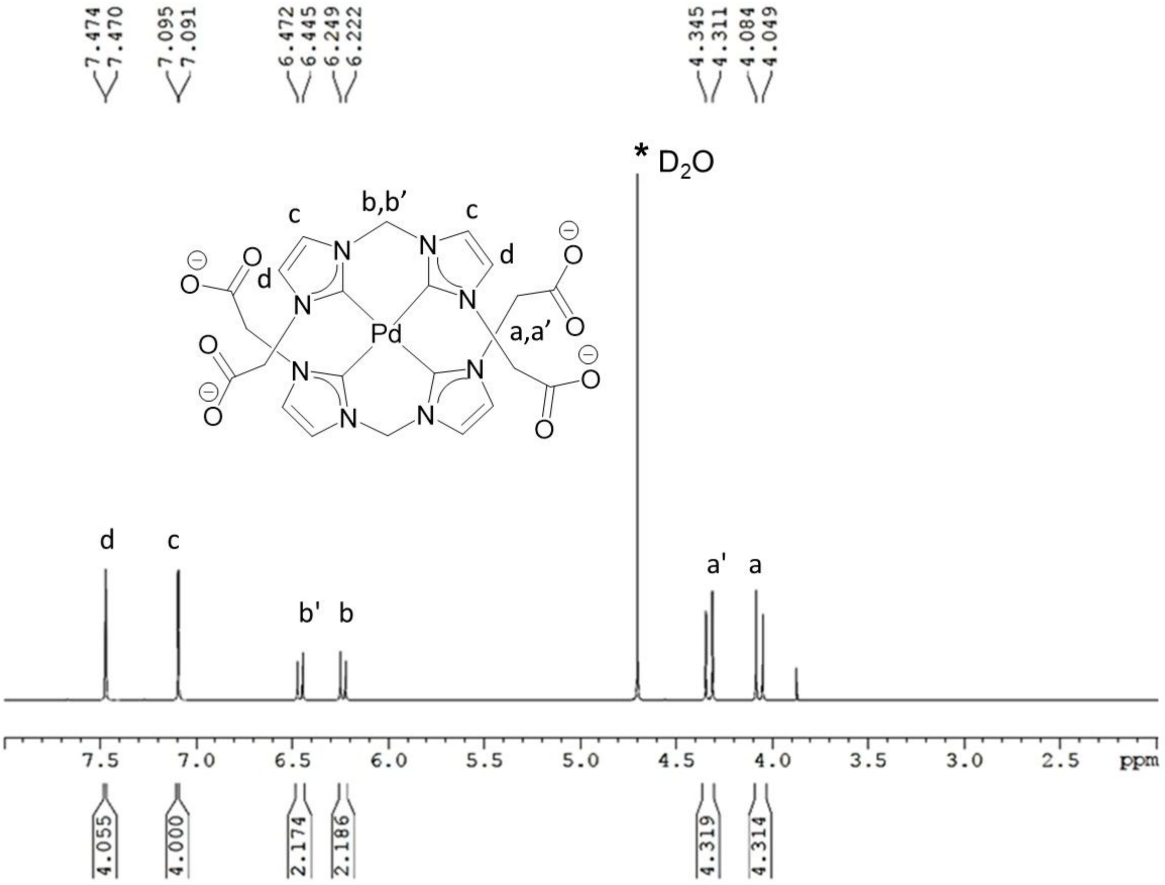
7.474
7.470
7.095
7.091
6.472
6.445
6.249
6.222
4.345
4.311
4.084
4.049
* D2O
b,b'
c
c
d
d
N
Pd
a,a'
d
c
b'
b
a'
a
7.5
7.0
6.5
6.0
5.5
5.0
4.5
4.0
3.5
3.0
2.5
ppm
4.055
4.000
2.174
2.186
4.319
4.314

$^{13}C\{^1H\}$ *NMR spectra*

1a

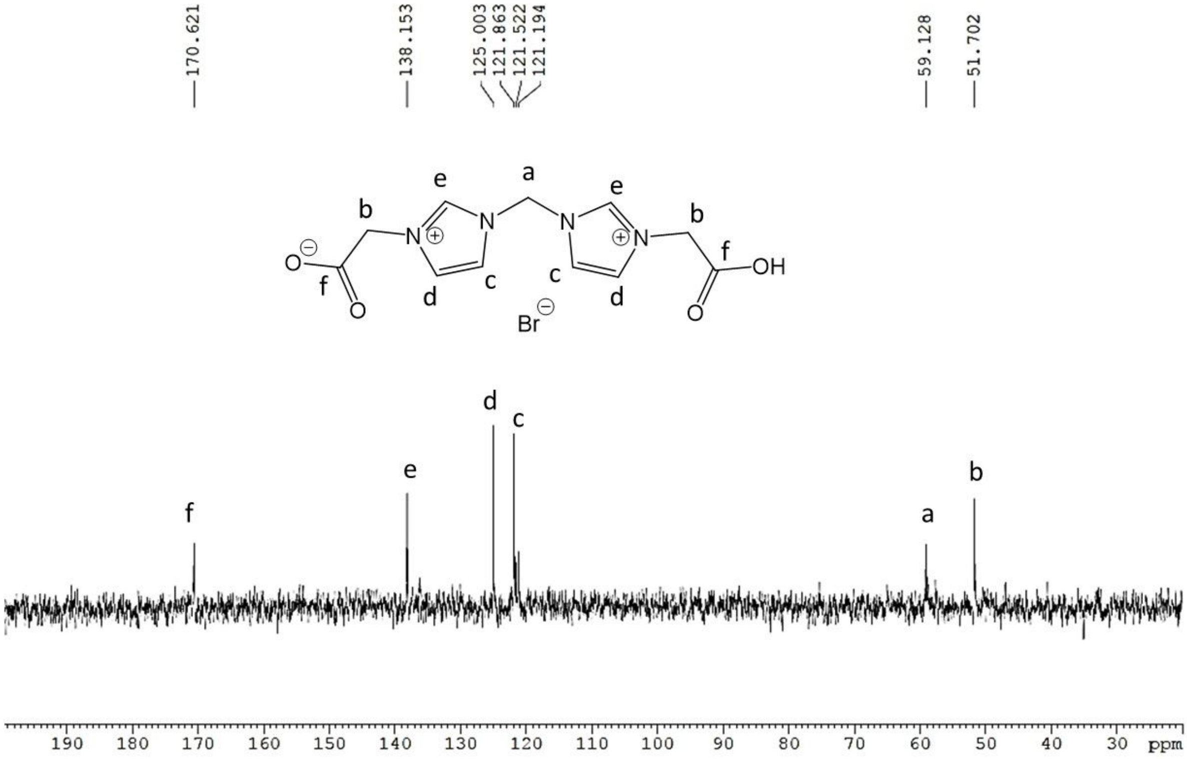

1b

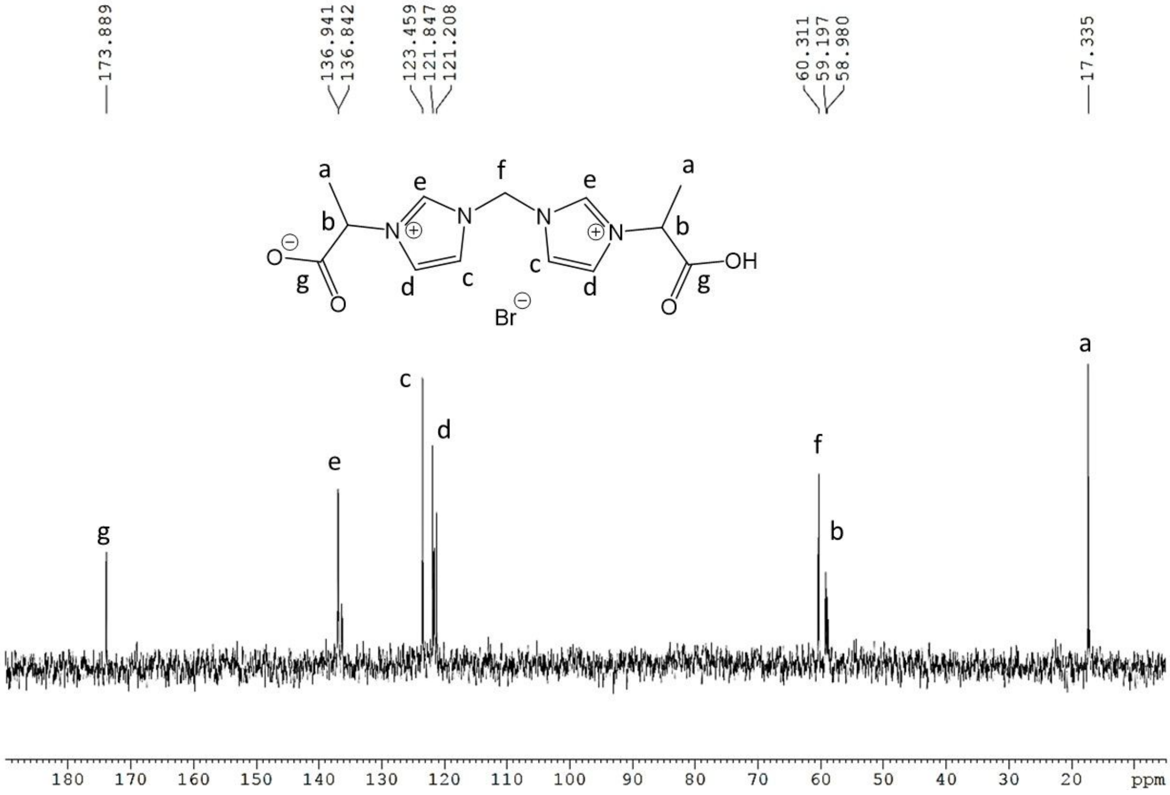

1c

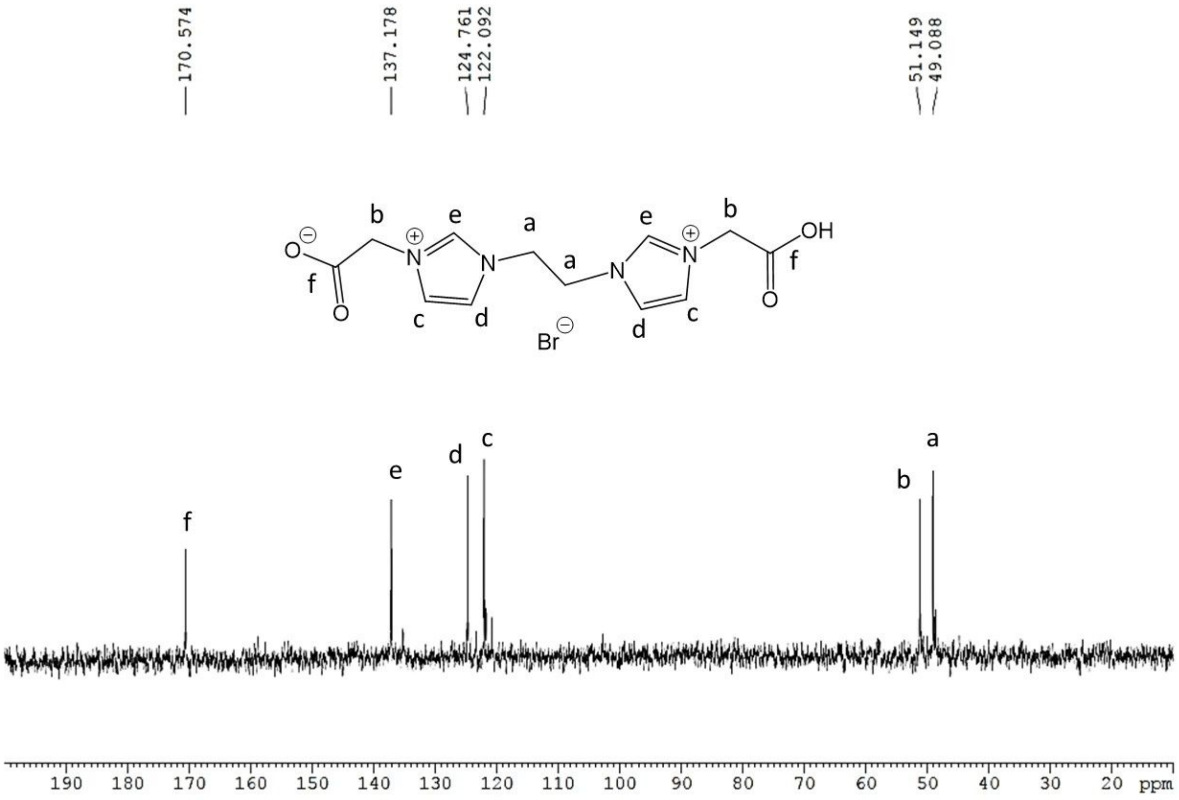
170.574
137.178
124.761
122.092
51.149
49.088
b
e
a
e
b
OH
f
a
f
O
c
d
Br
d
c
f
e
d
c
b
a
190
180
170
160
150
140
130
120
110
100
90
80
70
60
50
40
30
20
ppm

3a

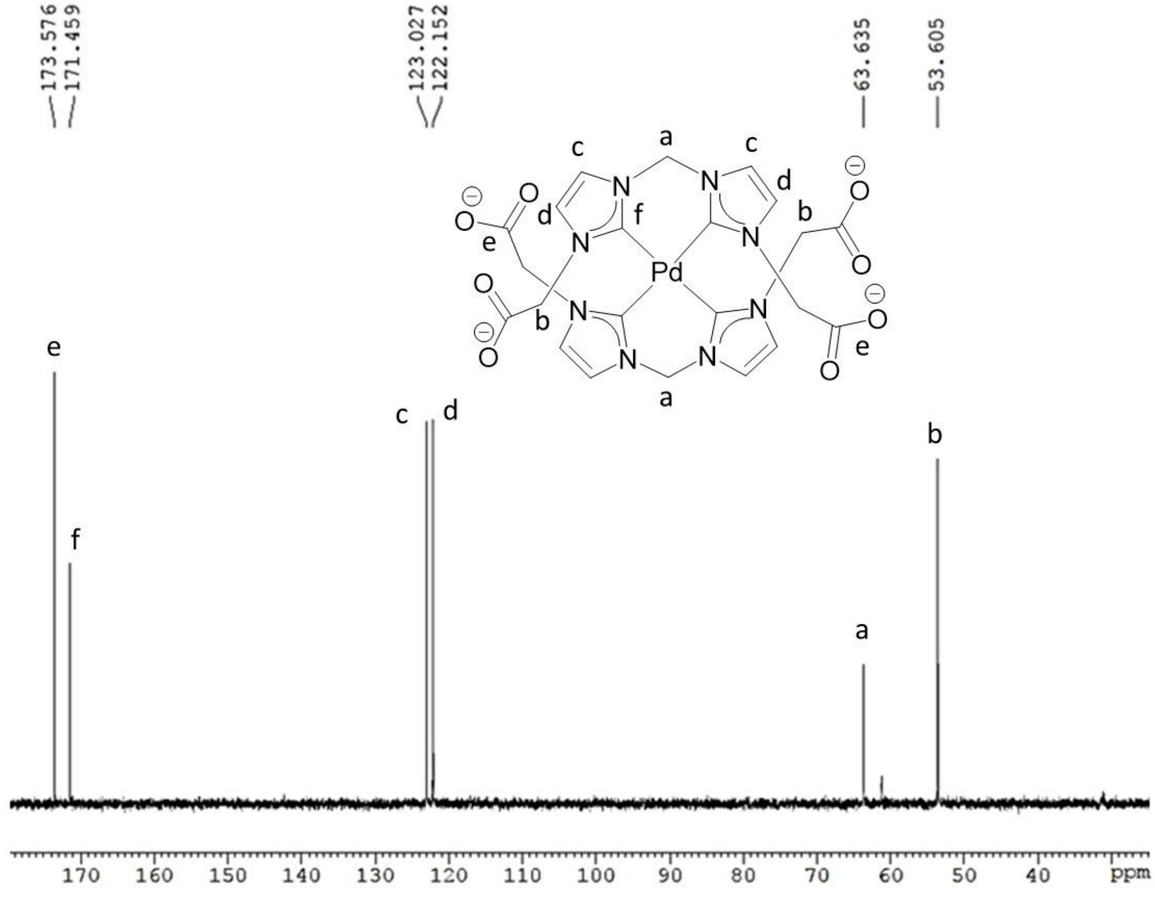
173.576
171.459
123.027
122.152
63.635
53.605
a
c
c
N
N
d
O
d
f
b
O
N
e
N
Pd
O
b
O
e
a
e
f
c
d
b
a
170
160
150
140
130
120
110
100
90
80
70
60
50
40
ppm

^{1}H-$^{13}C\{^{1}H\}$ *HSQC NMR spectra*

3a

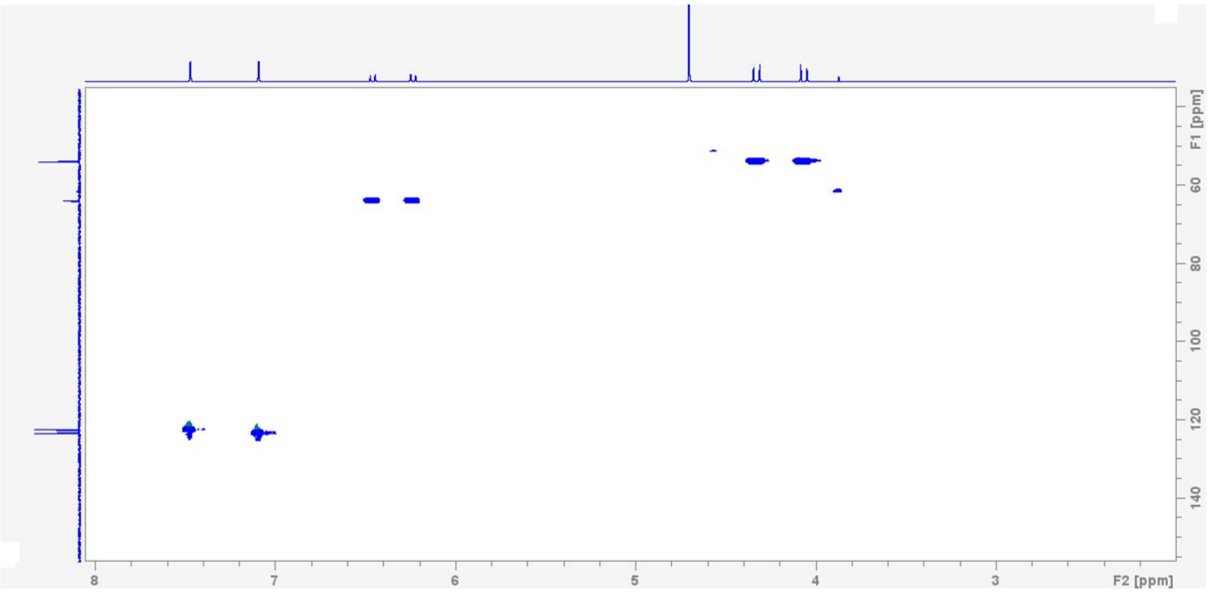

Table S1. Bond lengths and angles for **1a**.

**Atom**	**Atom**	**Length/Å**	**Atom**	**Atom**	**Length/Å**
O1	C6	1.223(7)	N2	C1	1.318(6)
O2	C6	1.237(7)	N2	C3	1.379(7)
N1	C1	1.317(6)	N2	C4	1.462(7)
N1	C2	1.366(6)	C2	C3	1.333(9)
N1	C5	1.466(6)	C5	C6	1.521(7)

**Atom**	**Atom**	**Atom**	**Angle/º**	**Atom**	**Atom**	**Atom**	**Angle/º**
C1	N1	C2	108.8(4)	C3	C2	N1	107.3(5)
C1	N1	C5	125.0(4)	C2	C3	N2	107.0(5)
C2	N1	C5	126.1(4)	N2[1]	C4	N2	108.6(7)
C1	N2	C3	108.3(4)	N1	C5	C6	113.9(4)
C1	N2	C4	124.5(4)	O1	C6	O2	127.4(5)
C3	N2	C4	127.2(4)	O1	C6	C5	113.8(5)
N1	C1	N2	108.7(4)	O2	C6	C5	118.8(5)

[1] 1/2-X, 1/2-Y, +Z

Table S2. Bond lengths and angles for **1b**.

**Atom**	**Atom**	**Length/Å**	**Atom**	**Atom**	**Length/Å**
O2	C6	1.195(6)	N2	C3	1.378(6)
O1	C6	1.290(6)	N2	C4	1.456(5)
N1	C1	1.326(6)	C2	C3	1.335(7)
N1	C2	1.376(6)	C5	C6	1.544(7)
N1	C5	1.481(5)	C5	C7	1.509(8)
N2	C1	1.342(6)			

**Atom**	**Atom**	**Atom**	**Angle/º**	**Atom**	**Atom**	**Atom**	**Angle/º**
C1	N1	C2	109.4(4)	C2	C3	N2	107.5(4)
C1	N1	C5	127.0(4)	N2	C4	N2[1]	112.4(5)
C2	N1	C5	123.4(4)	N1	C5	C6	109.9(4)
C1	N2	C3	108.5(4)	N1	C5	C7	111.2(4)
C1	N2	C4	124.7(4)	C7	C5	C6	110.6(4)
C3	N2	C4	126.7(4)	O2	C6	O1	125.8(5)
N1	C1	N2	107.6(4)	O2	C6	C5	118.9(5)
C3	C2	N1	107.1(4)	O1	C6	C5	115.2(4)

[1] +X, 1/2-Y, +Z

Table S3. Crystal data and structure refinement for **1a** and **1b**.

	**1a**	**1b**
Empirical formula	$C_{11}H_{13}N_4O_4Br$	$C_{13}H_{17}N_4O_4Br$
Formula weight	345.16	373.21
Temperature/K	193.00	193.00
Crystal system	orthorhombic	orthorhombic
Space group	Pccn	Pnma
a/Å	20.103(4)	12.6703(18)
b/Å	6.7804(12)	23.849(4)
c/Å	9.7499(16)	4.9757(9)
α/°	90	90
β/°	90	90
γ/°	90	90
Volume/Å^3	1329.0(4)	1503.5(4)
Z	4	4
ρ_{calc}g/cm^3	1.725	1.649
μ/mm^{-1}	3.113	2.758
F(000)	696.0	760.0
Crystal size/mm^3	0.5 × 0.3 × 0.05	0.25 × 0.1 × 0.05
Radiation	MoKα (λ = 0.71073)	MoKα (λ = 0.71073)
2Θ range for data collection/°	4.052 to 50.488	6.432 to 50.494
Index ranges	$-24 \le h \le 24$, $-7 \le k \le 8$, $-11 \le l \le 11$	$-14 \le h \le 15$, $-28 \le k \le 28$, $-5 \le l \le 5$
Reflections collected	11645	13142
Independent reflections	1199 [R_{int} = 0.0594, R_{sigma} = 0.0374]	1387 [R_{int} = 0.0686, R_{sigma} = 0.0318]
Data/restraints/parameters	1199/0/92	1387/0/104
Goodness-of-fit on F^2	1.201	1.148
Final R indexes [I>=2σ (I)]	R_1 = 0.0623, wR_2 = 0.1580	R_1 = 0.0584, wR_2 = 0.1643
Final R indexes [all data]	R_1 = 0.0653, wR_2 = 0.1597	R_1 = 0.0619, wR_2 = 0.1670
Largest diff. peak/hole / e Å^{-3}	1.31/-0.59	1.51/-0.86

Figure S2. Structures of compounds **1a** (top) and **1b** (bottom) and their structural description.

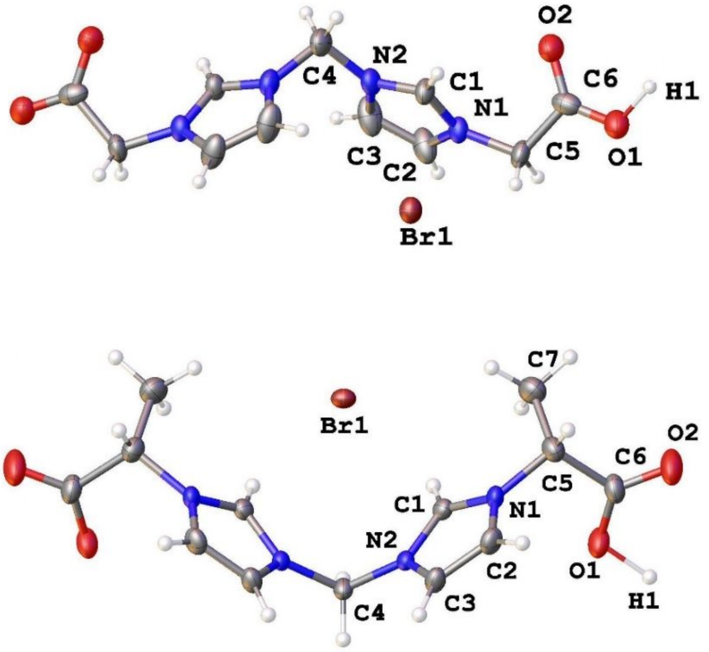

The carboxylate C=O distances showed slight asymmetry, more pronounced in **1b** (O2-C6, 1.195(6) and O1-C6, 1.290(6) Å). The N-C_{bridge}-N bond angle is slightly larger in **1b** (112.4(5)°) than in **1a** (108.6(7)°). Other bond distances and angles are typical and do not require further discussion. The angles between the planes of the imidazolium rings are 81.96 for **1a** and 67.49° for **1b**, while the planes defined by the carboxylate groups are nearly parallel (3.40 for **1a** and 9.03° for **1b**). Both **1a** and **1b** form strong intermolecular hydrogen bonds with neighboring molecules, significantly influencing their crystal packing. These hydrogen bonding interactions result in 1D polymeric chains that extend along *a* axis in **1a** and the *b* axis in **1b** (Figures S3a and S3b, respectively).

Figure S3. Compound **1a**: (up) polymeric chain viewed along *b* axis made up by hydrogen bonds; (b) crystal packing viewed along *b* axis. Color codes: C, grey; H, white; N, blue; O, red; Br, dark red.

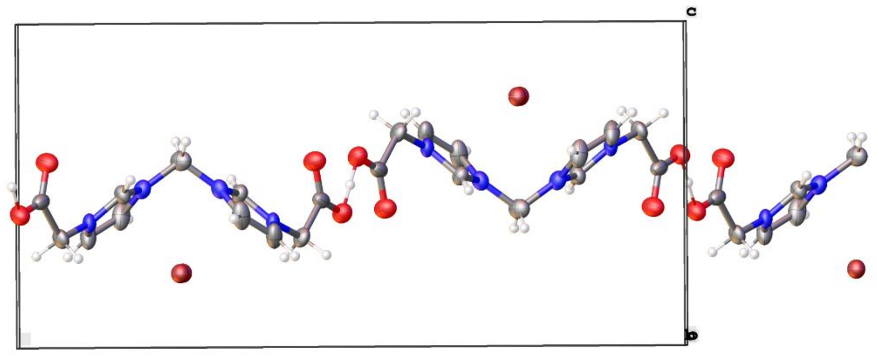

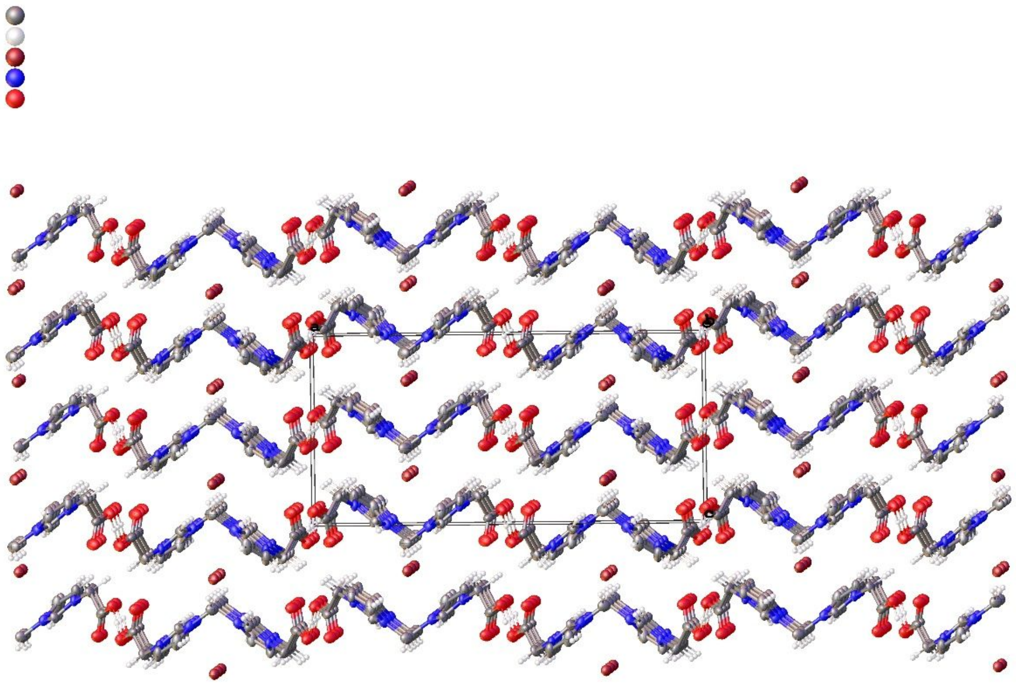

Figure S4. Compound **1b**: (up) polymeric chain viewed along *a* axis made up by hydrogen bonds; (b) crystal packing viewed along *c* axis. Color codes: C, grey; H, white; N, blue; O, red; Br, dark red.

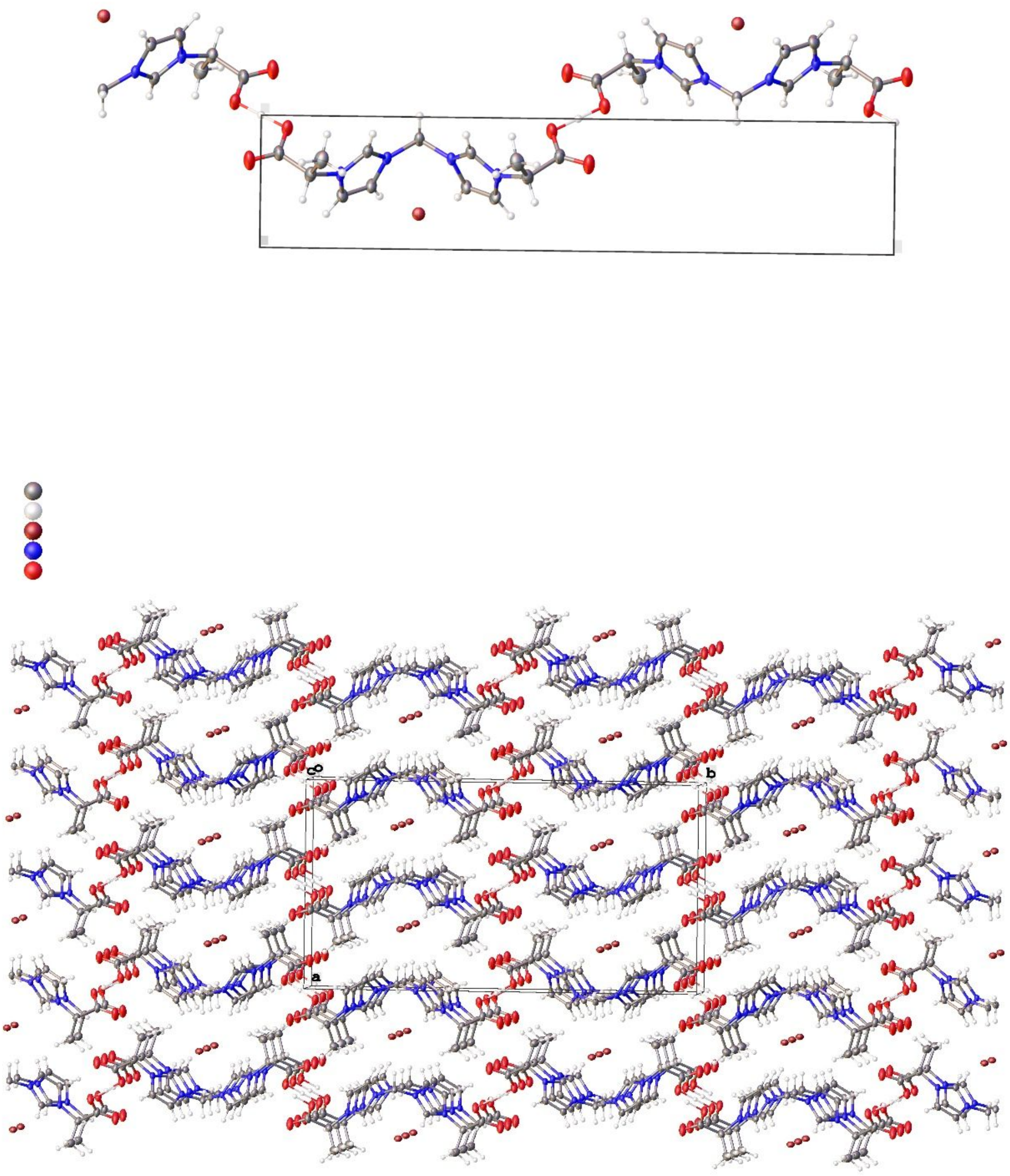

Table S4. Selected bond lengths and angles for **2a**.

Atom	Atom	Length/Å	Atom	Atom	Length/Å
Ag1	Ag2	3.3794(9)	O8	C22	1.259(11)
Ag1	C1	2.078(8)	Na1	O1	2.621(8)
Ag1	C12	2.085(8)	Na1	O5	2.343(7)
Ag2	C7	2.110(8)	Na1	O9	2.395(7)
Ag2	C18	2.106(8)	Na1	O10	2.451(7)
O1	C5	1.250(10)	Na1	O11	2.402(7)
O2	C5	1.256(11)	Na2	O10	2.521(8)
O3	C11	1.244(11)	Na2	O12	2.374(8)
O4	C11	1.250(10)	Na2	O13	2.395(8)
O5	C16	1.241(10)	Na2	O14	2.551(8)
O6	C16	1.247(10)	Na2	O15	2.910(11)
O7	C22	1.231(11)	Na2	O16	2.432(8)

Atom	Atom	Atom	Angle/º	Atom	Atom	Atom	Angle/º
C1	Ag1	Ag2	94.2(2)	O3	C11	O4	127.8(8)
C1	Ag1	C12	166.4(3)	O3	C11	C10	118.8(7)
C12	Ag1	Ag2	98.2(2)	O5	C16	O6	127.7(8)
C7	Ag2	Ag1	95.2(2)	O5	C16	C15	113.7(7)
C18	Ag2	Ag1	94.4(2)	O6	C16	C15	118.6(7)
C18	Ag2	C7	166.9(3)	O7	C22	O8	126.9(8)
O1	C5	O2	127.2(8)	O7	C22	C21	114.4(8)
O1	C5	C4	114.5(8)	O8	C22	C21	118.6(8)
O2	C5	C4	118.4(7)				

Table S5. Bond lengths and angles for **3a**.

Atom	Atom	Length/Å	Atom	Atom	Length/Å
Pd1	C1	2.0305(11)	N1	C2	1.3860(16)
Pd1	C1[1]	2.0305(11)	N1	C8	1.4576(16)
Pd1	C5[1]	2.0200(11)	N2	C1	1.3576(15)
Pd1	C5	2.0200(11)	N2	C3	1.3884(16)
Na1	Na1[2]	3.4893(11)	N2	C4	1.4564(16)
Na1	O5	2.5213(13)	N3	C5	1.3429(15)
Na1	O6	2.4437(12)	N3	C6	1.3907(16)
Na1	O6[2]	2.4271(12)	N3	C10	1.4546(15)
Na1	O7	2.3889(12)	N4	C4	1.4602(16)
Na1	O8	2.3973(12)	N4	C5	1.3548(15)
Na1	O9	2.4019(13)	N4	C7	1.3871(16)
O1	C9	1.232(2)	C2	C3	1.3530(19)
O2	C9	1.237(2)	C6	C7	1.3506(19)
O3	C11	1.2465(16)	C8	C9	1.5299(18)
O4	C11	1.2574(15)	C10	C11	1.5260(17)
N1	C1	1.3471(15)			

[1]1-X, 1-Y, 2-Z; [2]-X, -Y, 1-Z

Atom	Atom	Atom	Angle/°	Atom	Atom	Atom	Angle/°
C1	Pd1	C1[1]	180.0	C2	N1	C8	124.27(11)
C5[1]	Pd1	C1[1]	82.88(5)	C1	N2	C3	111.33(10)
C5	Pd1	C1	82.88(5)	C1	N2	C4	121.88(10)
C5	Pd1	C1[1]	97.12(5)	C3	N2	C4	126.40(11)
C5[1]	Pd1	C1	97.12(5)	C5	N3	C6	111.10(10)
C5[1]	Pd1	C5	180.00(4)	C5	N3	C10	123.25(10)
O5	Na1	Na1[2]	81.99(3)	C6	N3	C10	125.41(10)
O6	Na1	Na1[2]	44.05(3)	C5	N4	C4	121.40(10)
O6[2]	Na1	Na1[2]	44.43(3)	C5	N4	C7	111.03(10)
O6[2]	Na1	O5	88.95(4)	C7	N4	C4	126.86(11)
O6	Na1	O5	79.58(4)	N1	C1	Pd1	132.62(9)
O6[2]	Na1	O6	88.49(4)	N1	C1	N2	104.72(10)
O7	Na1	Na1[2]	132.52(4)	N2	C1	Pd1	122.36(8)
O7	Na1	O5	112.65(4)	C3	C2	N1	107.20(11)
O7	Na1	O6[2]	89.34(4)	C2	C3	N2	105.83(11)
O7	Na1	O6	167.54(5)	N2	C4	N4	108.53(10)
O7	Na1	O8	83.52(4)	N3	C5	Pd1	131.88(9)
O7	Na1	O9	94.45(4)	N3	C5	N4	104.90(10)
O8	Na1	Na1[2]	81.67(3)	N4	C5	Pd1	123.02(9)
O8	Na1	O5	162.35(5)	C7	C6	N3	106.59(11)
O8	Na1	O6[2]	84.05(4)	C6	C7	N4	106.38(11)
O8	Na1	O6	84.05(4)	N1	C8	C9	112.92(11)
O8	Na1	O9	97.00(4)	O1	C9	O2	125.09(16)

Atom	Atom	Atom	Angle/º	Atom	Atom	Atom	Angle/º
O9	Na1	Na1[2]	131.98(4)	O1	C9	C8	115.96(15)
O9	Na1	O5	89.00(4)	O2	C9	C8	118.94(14)
O9	Na1	O6[2]	176.15(4)	N3	C10	C11	115.02(10)
O9	Na1	O6	87.94(4)	O3	C11	O4	126.15(12)
Na1[2]	O6	Na1	91.51(4)	O3	C11	C10	119.03(11)
C1	N1	C2	110.92(10)	O4	C11	C10	114.76(11)
C1	N1	C8	124.46(10)				

[1]1-X, 1-Y, 2-Z; [2]-X, -Y, 1-Z

Table S6. Crystal data and structure refinement for **2a** and **3a**.

Complex	**2a**	**3a**
Empirical formula	$C_{44}H_{88}Ag_4N_{16}Na_4O_{40}$	$C_{22}H_{52}N_8O_{24}PdNa_2$
Formula weight	2004.74	965.09
Temperature/K	193.00	193.00
Crystal system	monoclinic	triclinic
Space group	$P2_1/c$	P-1
a/Å	14.492(2)	8.9069(5)
b/Å	21.754(3)	10.7294(6)
c/Å	12.3949(15)	11.0925(6)
α/°	90	96.088(2)
β/°	109.378(5)	100.908(2)
γ/°	90	102.775(2)
Volume/Å^3	3686.1(8)	1002.96(10)
Z	2	1
ρ_{calc}g/cm^3	1.806	1.598
μ/mm^{-1}	1.176	0.580
F(000)	2032.0	500.0
Crystal size/mm^3	0.3 × 0.2 × 0.05	0.5 × 0.45 × 0.35
Radiation	MoKα (λ = 0.71073)	MoKα (λ = 0.71073)
2Θ range for data collection/°	3.954 to 50.492	3.784 to 61.084
Index ranges	$-16 \leq h \leq 17$, $-24 \leq k \leq 26$, $-14 \leq l \leq 14$	$-12 \leq h \leq 12$, $-15 \leq k \leq 15$, $-15 \leq l \leq 15$
Reflections collected	36839	54461
Independent reflections	6667 [R_{int} = 0.0648, R_{sigma} = 0.0448]	6062 [R_{int} = 0.0425, R_{sigma} = 0.0287]
Data/restraints/parameters	6667/120/516	6062/72/276
Goodness-of-fit on F^2	1.084	1.115
Final R indexes [I>=2σ (I)]	R_1 = 0.0669, wR_2 = 0.1598	R_1 = 0.0261, wR_2 = 0.0675
Final R indexes [all data]	R_1 = 0.0823, wR_2 = 0.1658	R_1 = 0.0262, wR_2 = 0.0676
Largest diff. peak/hole / e Å^{-3}	2.59/-1.70	0.68/-0.86

Figure S5. Front and side views of the anion of **2a**. Color codes: C, grey; H, white; Ag, light grey; N, blue; O, red.

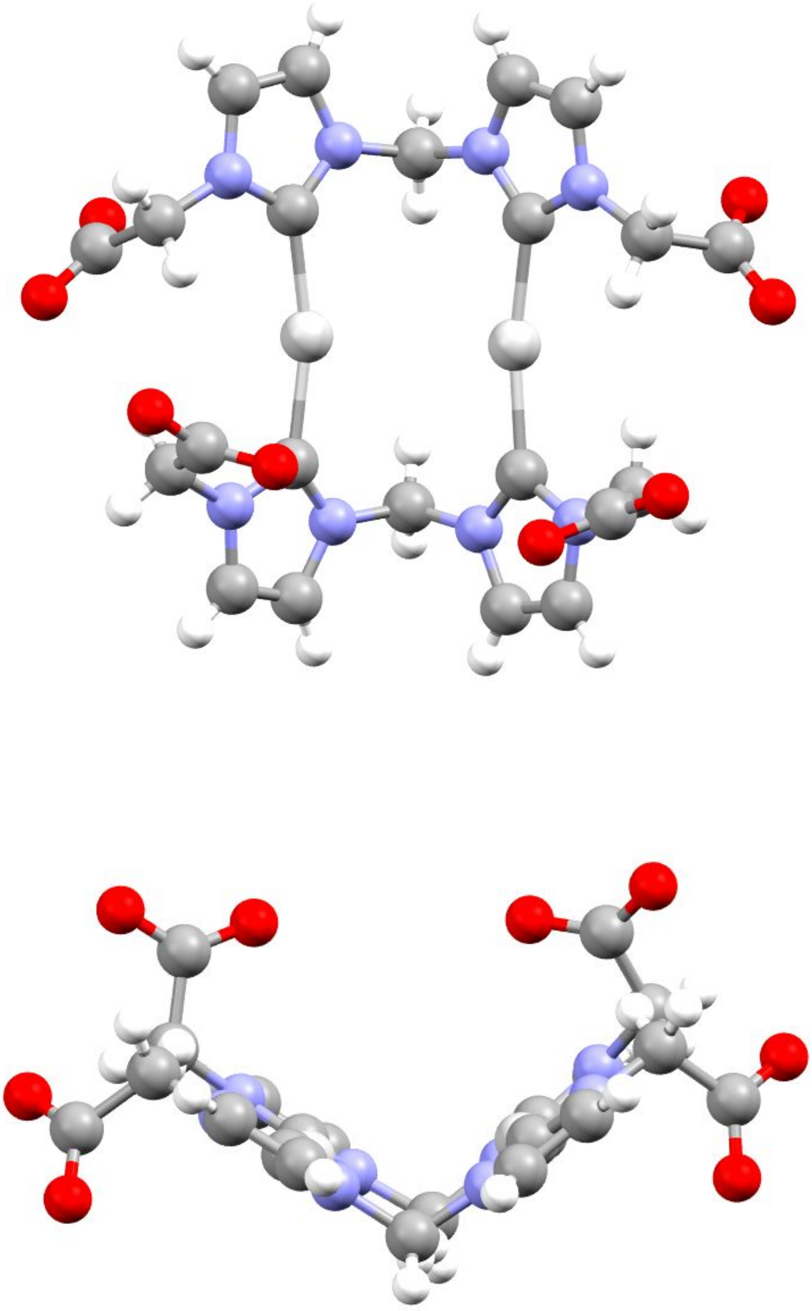

Figure S6. Crystal packing of **2a** along *a*, *b* and *c* axis. Color codes: C, grey; H, white; Ag, cyan; N, blue; Na, light blue; O, red.

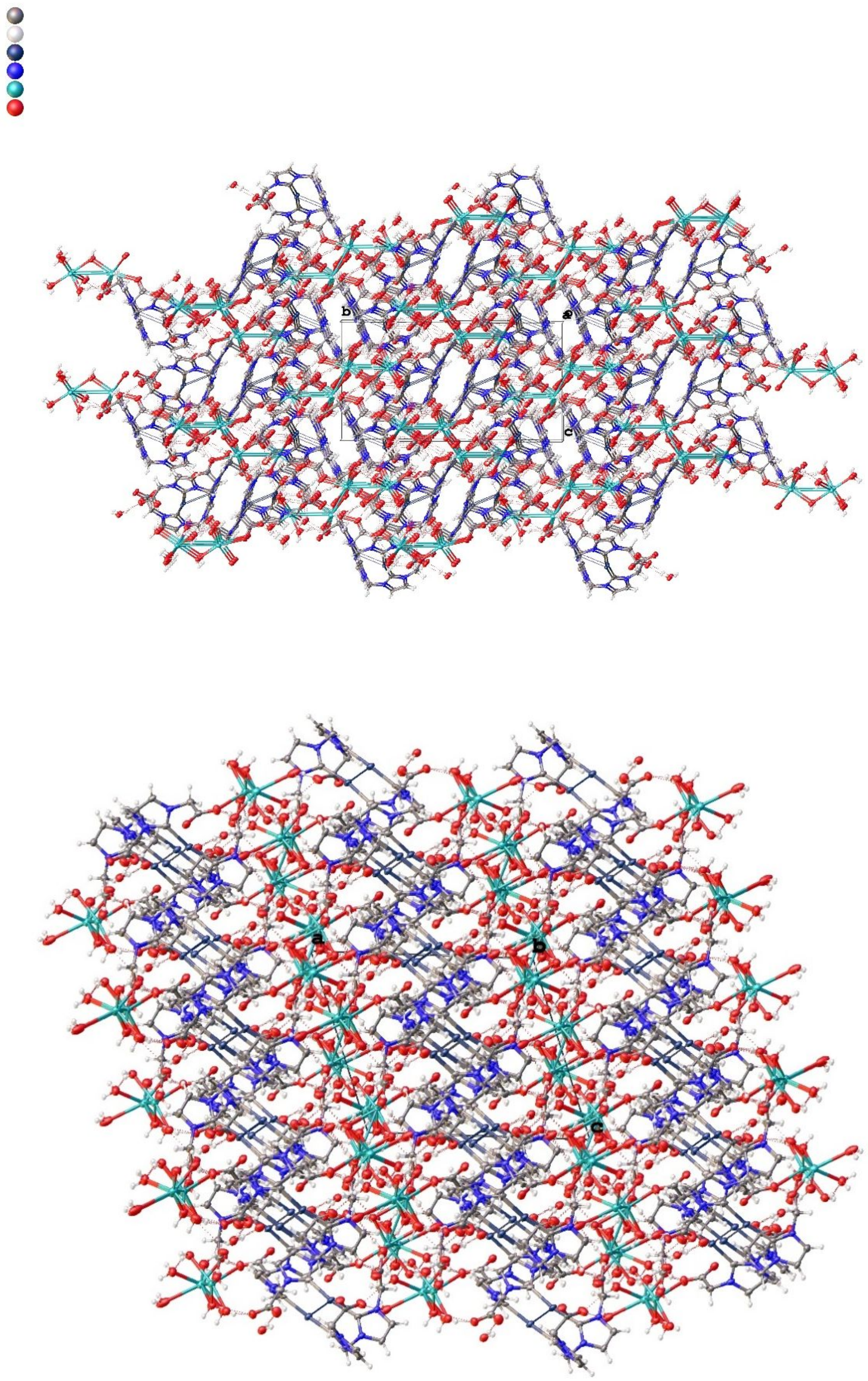

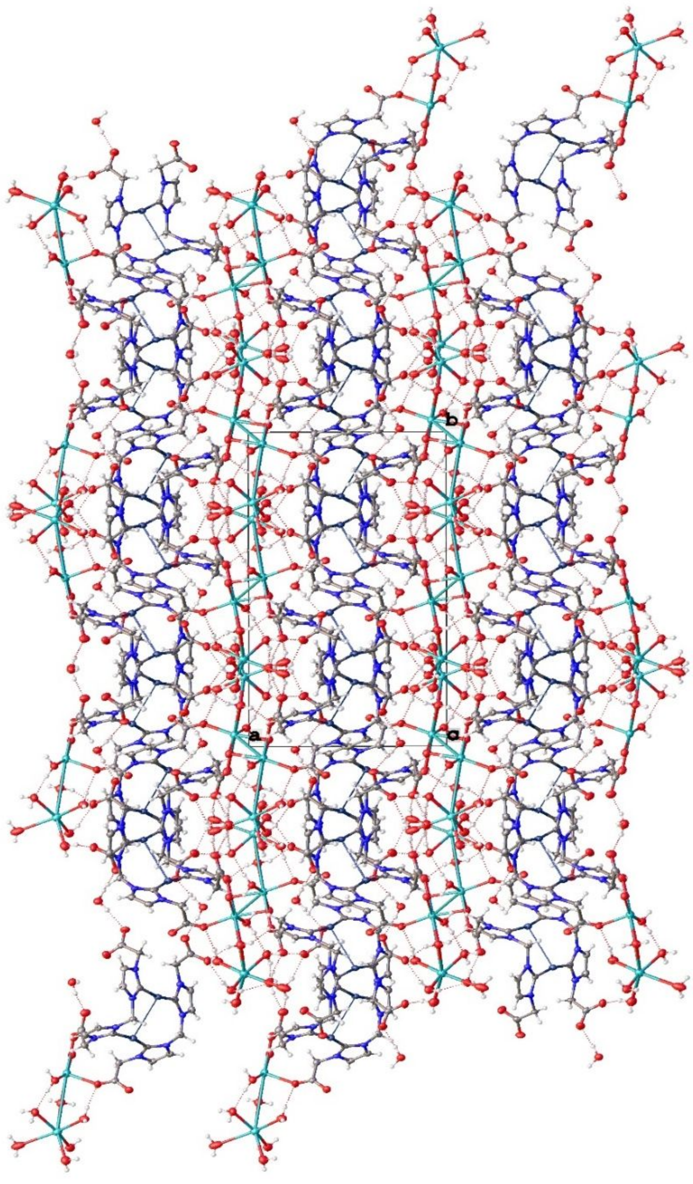
b
a
o

Figure S7. Crystal packing of **3a** along *a* axis. Color codes: C, grey; H, white; Pd, blue-gray; N, blue; Na, light blue; O, red.

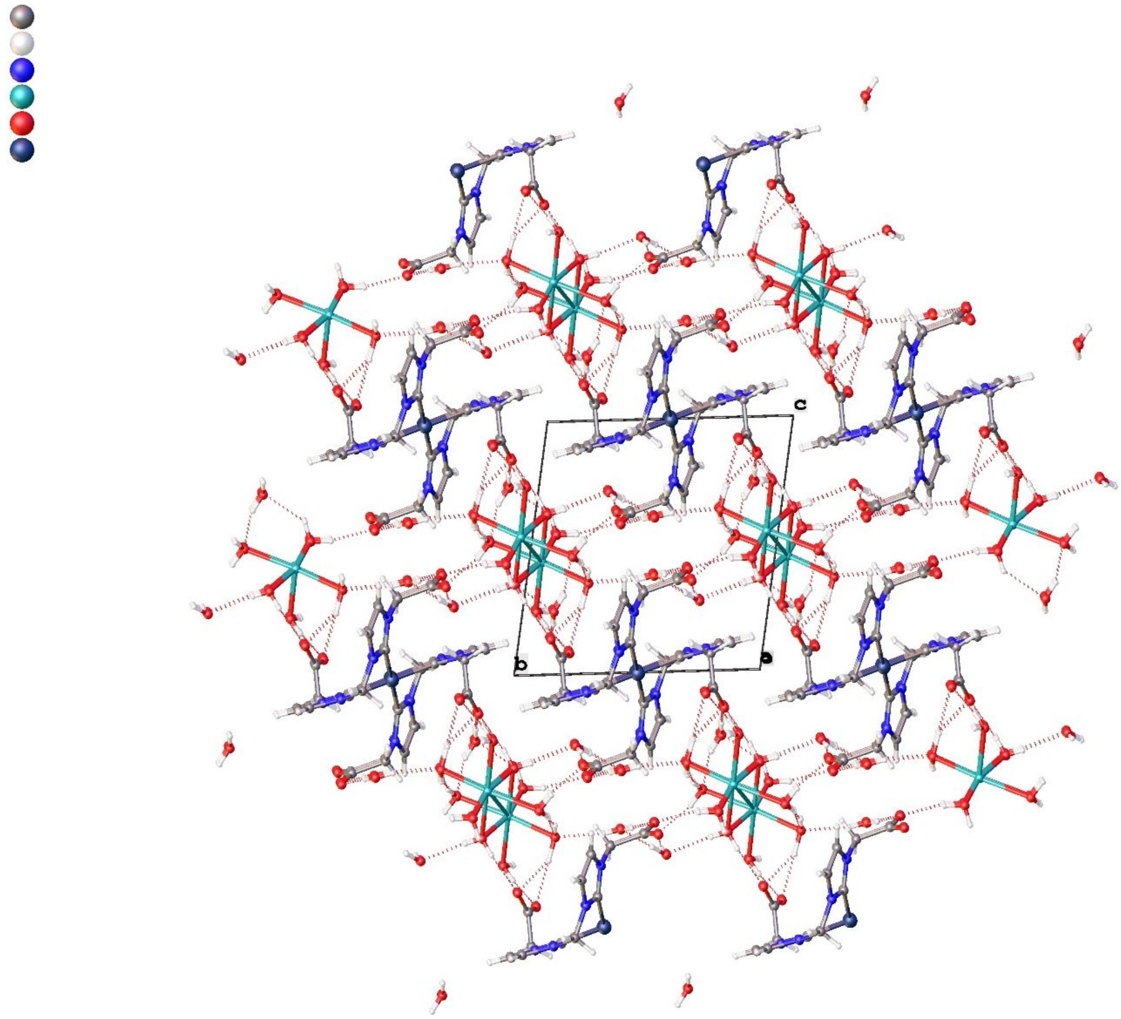

DFT protocol for estimating HEP2 values.

The following protocol describes the procedure for estimating the HEP2 value of a bidentate ligand (L_2) via DFT calculations:

1. Optimize the structure of the complex $[PdBr(L_2)(^iPr_2\text{-bimy})]^{n+}$ ($^iPr_2$-bimy = 1,3-diisopropylbenzimidazolin-2-ylidene n = -1, 0, 1 for a dianionic, anionic or neutral bidentate ligand, respectively;). Functional mpw1pw91 was used with the LANL2DZ basis set for palladium and the 6-31G(d,p) basis set for other atoms.

   Representative input file for $L_2$1 (bipy):

```
%mem=64GB
%nprocshared=28
%chk=HEP2_Pd_bipy.chk
# mpw1pw91/gen pseudo=read opt freq test

Model HEP2 with bipy ligand L2-1

1 1
 Pd              -0.82323700   -0.37337700   -0.03178300
 Br              -0.12600800   -2.69954200   -0.10292400
 C                3.22729500    0.28747700    0.74082500
 C                3.24811900    0.35436900   -0.65992800
 C                4.43687900    0.58068000   -1.35454500
 C                5.59272100    0.73716700   -0.60423700
 C                5.57034400    0.67534100    0.79668000
 C                4.39154600    0.45182100    1.49188300
 C                1.14176100   -0.02862700   -0.00590800
 H                4.47246200    0.62513300   -2.43488400
 H                6.53363200    0.90848400   -1.11406600
 H                6.49389200    0.80161200    1.34969500
 H                4.39028200    0.40220100    2.57274600
 N                1.90272700    0.05261600    1.10290100
 N                1.93351600    0.16209300   -1.08335500
 C                1.38314400   -0.16853700    2.46638500
 H                0.31452400   -0.34410500    2.31100700
 C                1.97980600   -1.43098200    3.07794400
 H                1.78975100   -2.29104800    2.43311800
 H                3.05579100   -1.34445000    3.23945600
 H                1.51141300   -1.61622400    4.04727100
 C                1.54594800    1.07580200    3.33401400
 H                1.04627700    0.91359000    4.29169500
 H                2.59227200    1.30472100    3.54422400
 H                1.09407500    1.95079100    2.86018400
 C                1.47029600    0.02939500   -2.47984600
 H                0.39428200   -0.14191900   -2.38155100
 C                1.67944900    1.31866600   -3.26786600
 H                1.20523600    2.17056500   -2.77401200
 H                2.73438100    1.55558500   -3.41432900
 H                1.22891800    1.20873000   -4.25686400
 C                2.08931900   -1.19702400   -3.14417400
 H                3.17212300   -1.10331900   -3.25062900
 H                1.86905000   -2.09502100   -2.56387400
 H                1.66486200   -1.31856400   -4.14363600
 C               -1.14365500    4.00778100    0.07231600
 C               -2.52201800    4.17775000    0.06459700
 C               -3.33963100    3.05290000    0.04285100
 C               -2.77293900    1.77925600    0.02657300
 C               -0.63898500    2.71271100    0.05422400
 C               -3.58588000    0.53763400    0.01129500
 C               -4.97686200    0.52488900    0.02902100
 C               -5.64894800   -0.69526300    0.02521600
 H               -6.73231700   -0.71334900    0.03910900
 C               -4.91906000   -1.87935800    0.00292300
 C               -3.53211200   -1.80068700   -0.01965200
 H               -0.46299900    4.85037900    0.09162600
 H               -2.96051300    5.17021100    0.07607800
```

```
 H                 0.42779100    2.52737200    0.06127900
 H                -5.40213100   -2.84989500    0.00080200
 H                -2.89438600   -2.68012100   -0.04508400
 H                -5.54793300    1.45255900    0.04637900
 H                -4.41999000    3.17926900    0.03811800
 N                -2.89178500   -0.62605800   -0.01511600
 N                -1.42314100    1.62808600    0.02942600

Pd
LANL2DZ
****
C N H Br
6-31G(d,p)
****

Pd
LANL2DZ
```

2. Perform an NMR calculation on the optimized complex to determine the ^{13}C-NMR isotropic shielding tensors using the GIAO method. To take advantage of the TMS reference that appears in the Gauss View program, the NMR calculation was performed at the B3LYP level with the LANL2DZ basis set for palladium and the 6-311+G(2d,p) basis set for other atoms.

   Representative input file for $L_2$1 (bipy):

```
%mem=64Gb
%nproc=28
%chk=NMR_ HEP2_Pd_bipy.chk
#T B3LYP/gen pseudo=read NMR scrf=(cpcm) Guess=Read Test

Model bipy RMN

1 1
 Pd                0.79645300   -0.34898000    0.00066300
 Br                0.17759800   -2.69083900   -0.00023700
 C                -3.26260200    0.31153700   -0.70128200
 C                -3.26307700    0.31110400    0.70069500
 C                -4.44186300    0.49465500    1.42369300
 C                -5.61068000    0.67575800    0.69984700
 C                -5.61013500    0.67655100   -0.70189500
 C                -4.44078600    0.49599800   -1.42501100
 C                -1.16433900   -0.02029900    0.00028100
 H                -4.45919600    0.49108100    2.50492700
 H                -6.54463400    0.81679100    1.23106600
 H                -6.54364300    0.81834100   -1.23369300
 H                -4.45723400    0.49364400   -2.50626400
 N                -1.94070700    0.10877900   -1.09351600
 N                -1.94138900    0.10840800    1.09366400
 C                -1.43980700   -0.05786800   -2.47101100
 H                -0.36605100   -0.21981400   -2.33795000
 C                -2.02448100   -1.31050700   -3.11230100
 H                -1.80217900   -2.18624300   -2.50005600
 H                -3.10557500   -1.24184700   -3.24708300
 H                -1.57385000   -1.45557300   -4.09642800
 C                -1.63413800    1.21143000   -3.29277900
 H                -1.15637300    1.08512900   -4.26650800
 H                -2.68643900    1.43956600   -3.47013300
 H                -1.17734600    2.07376500   -2.80123100
 C                -1.44104000   -0.05723700    2.47147800
 H                -0.36704100   -0.21810900    2.33903300
 C                -1.63710700    1.21228700    3.29250700
 H                -1.18104300    2.07485800    2.80070300
 H                -2.68973400    1.43937800    3.46923200
 H                -1.15967300    1.08704800    4.26652900
 C                -2.02467100   -1.31013900    3.11321300
```

```
H                  -3.10595400   -1.24272800    3.24710200
H                  -1.80080300   -2.18607600    2.50182900
H                  -1.57465200   -1.45387200    4.09782300
C                   1.18899500    3.99793100   -0.00050000
C                   2.56825700    4.16398000   -0.00096100
C                   3.38230500    3.03971600   -0.00050600
C                   2.80831100    1.76961000    0.00033600
C                   0.67845100    2.70851800    0.00031900
C                   3.59856900    0.52290300    0.00044300
C                   4.99041100    0.49189900    0.00142900
C                   5.64293400   -0.73580600    0.00082600
H                   6.72616200   -0.77464600    0.00163700
C                   4.89120000   -1.90253000   -0.00081000
C                   3.50517800   -1.80237900   -0.00134600
H                   0.51419700    4.84468300   -0.00086200
H                   3.00770200    5.15469900   -0.00174800
H                  -0.38832700    2.52604000    0.00063000
H                   5.35918600   -2.87893200   -0.00149100
H                   2.85728200   -2.67260100   -0.00224100
H                   5.56679300    1.40734000    0.00280100
H                   4.45785700    3.15429900   -0.00101600
N                   2.88240700   -0.62192500   -0.00063500
N                   1.46233200    1.62381200    0.00066100

Pd
LANL2DZ
****
C N H Br
6-311+G(2d,p)
****

Pd
LANL2DZ
```

3. Using GaussView or similar software, extract the ^{13}C chemical shift corresponding to the carbene carbon atom of the $^{i}Pr_2$-bimy ligand. This value will be used in the HEP2 estimation and for correlation with experimental data.

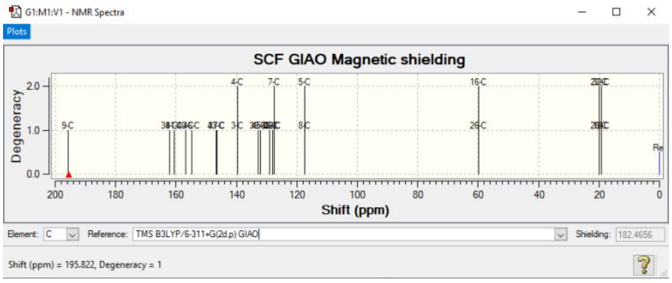

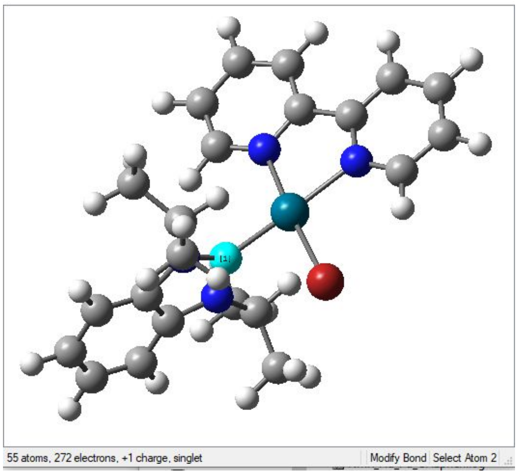

Figure S8. Optimized structures of bis(carbene)ligands.

di$_{CH2}$NHC^{H}

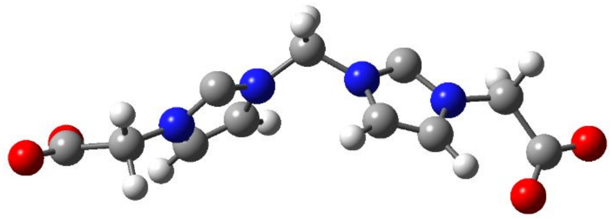

di$_{CH2}$NHC^{Me}

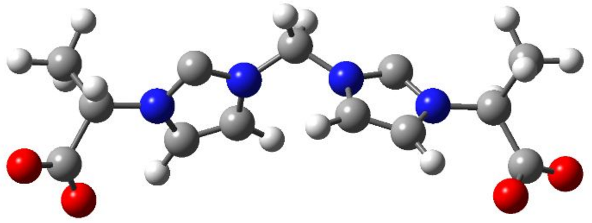

di$_{C2H4}$NHC^{H}

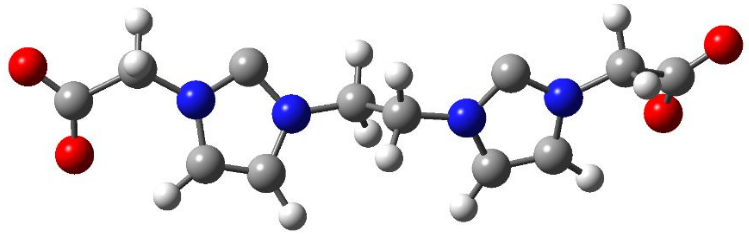

Table S7. Optimized structures of $[Ni(CO)_3(diNHC^R)]^{2-}$ complexes and selected calculated properties.

Complex	$diNHC^R$	Estimated TEP (cm^{-1})	Unscaled ν_{CO} (A_1)	d(Ni-C) (Å)	d(C-O) (Å)	Optimised structures[a]
$[Ni(CO)_3(diNHC^R)]^{2-}$	$di_{CH2}NHC^H$	2031.8	2129.5	2.007	1.145<br>1.149	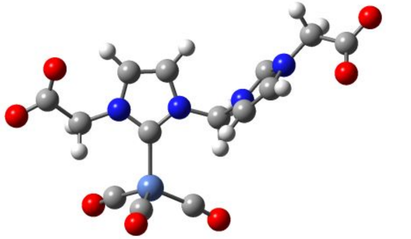
	$di_{CH2}NHC^{Me}$	2030.8	2128.5	2.011	1.145<br>1.147	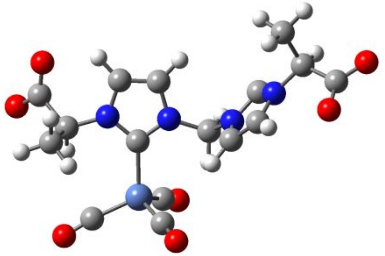
	$di_{C2H4}NHC^H$	2033.3	2131.1	2.001	1.145<br>1.148	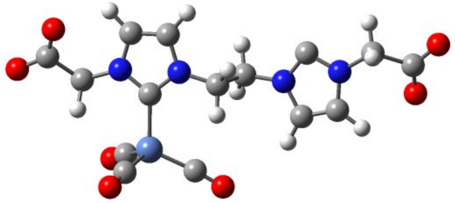

Table S8. Optimized structures of $[Ni(CO)_2(diNHC^R)]^{2-}$ complexes and selected calculated properties.

Complex	diNHC^R	Scaled ν_{CO} (cm^{-1}) [a]	Unscaled ν_{CO} (A_1)	d(Ni-C) (Å)	d(C-O) (Å)	Optimised structures[a]
	di$_{CH2}$NHC^H	1964.8	2046.7	1.990	1.154<br>1.159	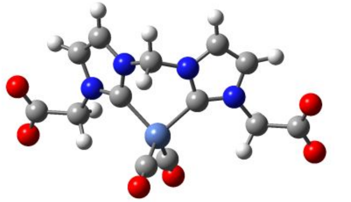
**$[Ni(CO)_2(diNHC^R)]^{2-}$**	di$_{CH2}$NHC^{Me}	1963.3	2045.1	1.993<br>1.994	1.154<br>1.160	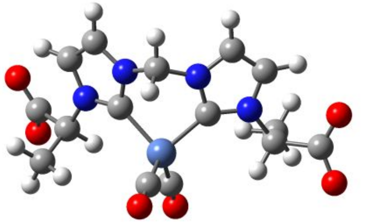
	di$_{C2H4}$NHC^H	1964.9	2046.8	1.994<br>2.001	1.156	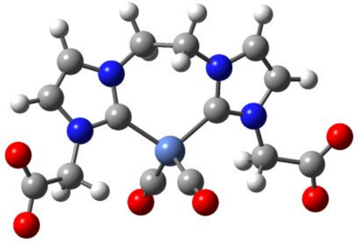

[a] Scaled carbonyl stretching frequencies by a factor of 0.96 (ref 23).

Table S9. Optimized structures of $[Mo(CO)_4(diNHC^R)]^{2-}$ complexes and selected calculated properties.

Complex	diNHC^R	Estimated TEP (cm^{-1}) [a]	Scaled ν_{CO} [b]	Unscaled ν_{CO} (A_1)	d(Mo-C) (Å)	d(C-O) (Å)	Optimised structures[a]
**$[Mo(CO)_4(diNHC^R)]^{2-}$**	di$_{CH2}$NHC^H	2061.0	2006.7	2090.3	2.285	1.149<br>1.158<br>1.159	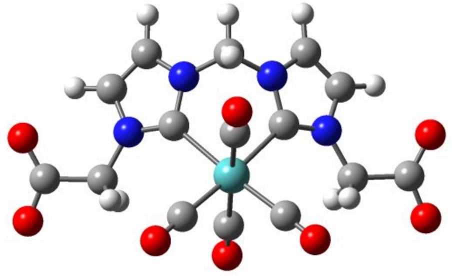
	di$_{CH2}$NHC^{Me}	2062.2	2008.7	2092.4	2.289<br>2.298	1.148<br>1.157<br>1.158<br>1-160	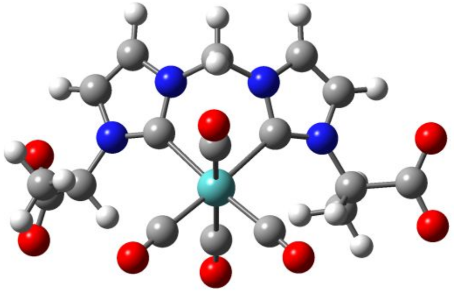
	di$_{C2H4}$NHC^H	2062.4	2009.1	2092.8	2.305<br>2.342	1.147<br>1.158<br>1.159	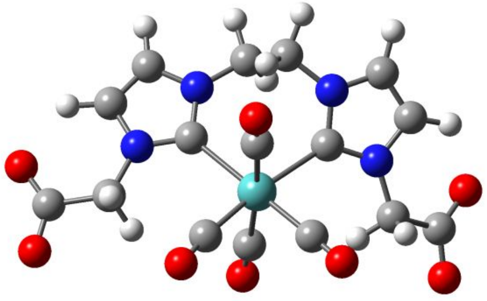

[a] Calculated using the equation: $\nu_{Ni} = 0.593\nu_{Mo} + 871$ (ref. 14). [b] Scaled carbonyl stretching frequencies by a factor of 0.96 (ref. 23).

Figure S9. Comparison of the calculated ^{13}C NMR chemical shifts using mpw1pw91 and B3LYP functionals.

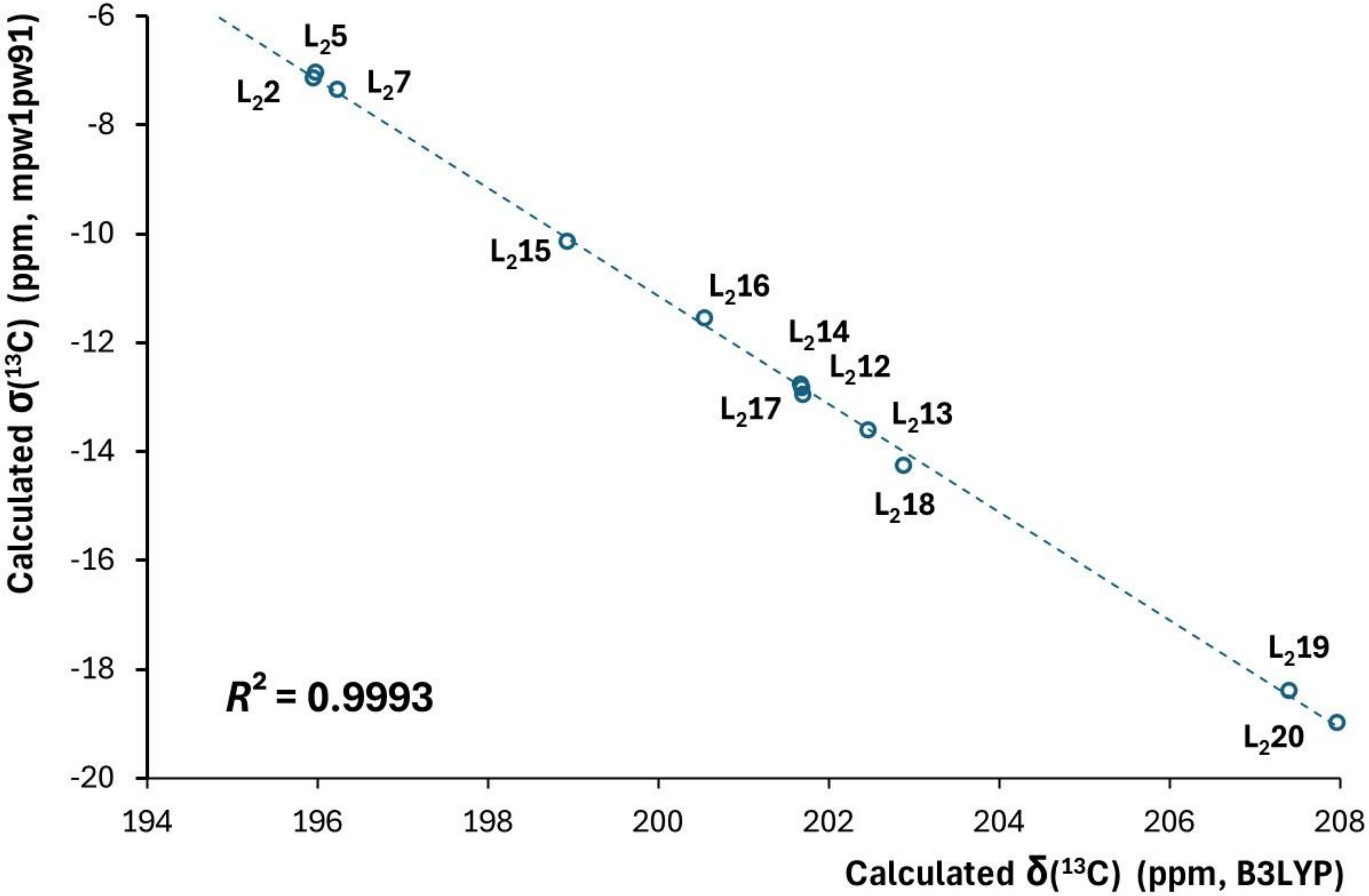

Supplement: Supplementary file 1 [file ic5c01231_si_001.pdf]
